# Health effects of street vended fresh cut fruits: A randomized controlled trial in Bangladesh

**DOI:** 10.1371/journal.pone.0335979

**Published:** 2025-10-31

**Authors:** F. N. U. Nahiduzzaman, Tasnim Zarin, Chandra Shaker Chouhan, Md. Zaminur Rahman, Mst. Minara Khatun, A. K. M. Anisur Rahman, Md. Ariful Islam, Md Azizul Haque

**Affiliations:** 1 Department of Microbiology and Hygiene, Bangladesh Agricultural University, Mymensingh, Bangladesh; 2 Department of Medicine, Faculty of Veterinary Science, Bangladesh Agricultural University, Mymensingh, Bangladesh; 3 Department of Health Informatics, Faculty of Medicine and Health Sciences, Frontier University, Puntland, Somalia; 4 Department of Biotechnology, Yeungnam University, Gyeongsan, Republic of Korea; Lusofona University of Humanities and Technologies: Universidade Lusofona de Humanidades e Tecnologias, PORTUGAL

## Abstract

Foodborne infections, particularly from street-vended fresh-cut fruits, are a growing public health concern in urban settings of developing countries. This study evaluated the gastrointestinal effects of consuming street-vended fruits in a randomized controlled trial (RCT) in Mymensingh, Bangladesh. A total of 300 participants were recruited and randomized into Treatment (n = 150) and Control (n = 150) groups. Treatment participants consumed guava, pineapple, or watermelon purchased from street vendors, while Control participants avoided street-vended fruits. Microbial analysis of fruits included total viable count (TVC), *S. aureus*, and *E. coli*. Participants recorded GI symptoms for 4 days post-intervention, with a 10-day follow-up. At least one GI symptom occurred in 41 (27.3%) treatment participants compared with 15 (10%) controls. Nausea affected 20 (13.3%) versus 2 (1.3%) participants (RR = 10, 95% CI: 2.38–42.03, p < 0.001), abdominal cramps 13 (8.7%) versus 0 (0%) participants (p < 0.001), and diarrhea 7 (4.7%) versus 0 (0%) participants (p = 0.02). Cox modeling indicated a markedly higher hazard of symptom development in the Treatment group (HR = 162.68, 95% CI: 21.53–1229.43, p < 0.001), while higher hygienic practice scores modestly reduced risk (HR = 0.90, 95% CI: 0.82–0.99, p = 0.028). Higher total viable counts (TVC 5.78–5.86 log CFU/ml) were strongly associated with weakness (r = 0.66, p < 0.001, OR=11.28), abdominal cramps (r = 0.56, p < 0.001, OR=6.16), and diarrhea (r = 0.51, p < 0.001, OR=6.73). *E. coli* (6–10% prevalence) showed the strongest correlations with abdominal cramps, weakness, and diarrhea (ρ = 0.69–0.78, p < 0.001), whereas *S. aureus* (20–34%) correlated primarily with weakness and abdominal cramps (ρ = 0.44–0.47, p < 0.001). Abdominal cramps, weakness, and diarrhea demonstrated the highest sensitivity to microbial contamination (AUC = 0.801–0.908). These findings provide robust evidence that consumption of street-vended fresh-cut fruits is associated with a significant increase in GI symptoms linked to microbial contamination, supporting behavioral interventions, such as consumer awareness and improved hygiene practices, and informing policy measures to enhance food safety and protect public health.

## 1 Introduction

In urban and peri-urban areas, especially in low- and middle-income countries, consuming fresh-cut fruits from street vendors is a common practice. These fruits provide a convenient and affordable source of essential nutrients. In Bangladesh, popular choices include guava, pineapple, and watermelon, valued for their affordability, nutritional benefits, and easy availability [1]. Despite their nutritional benefits, fresh-cut, street-vended fruits pose significant public health concerns as they often serve as vehicles for microbial contamination by pathogens that thrive in conditions typical of the street vending environment, such as the use of untreated water, inadequate refrigeration, and prolonged exposure to ambient temperatures [2,3]. The informal street food sector often faces challenges due to substandard hygienic practices during handling, preparation, storage, and serving, as well as exposure to environmental contaminants. This sector largely operates outside formal regulatory oversight [4]. These conditions foster an optimal environment for microbial growth, increasing the risks to consumers. Research consistently shows high levels of microbial contamination, including pathogenic organisms like *Salmonella spp.*, *E. coli*, and *S. aureus*, in fresh-cut street-vended foods [5]. In Bangladesh, *S. aureus* and *E. coli* with elevated loads were frequently isolated in fresh vegetables and street foods, indicating contamination risks during harvesting, handling, and unhygienic conditions [6,7]. *S. aureus* can cause gastrointestinal disturbances primarily through the production of enterotoxins, which are heat-stable and can survive cooking processes [8]. These toxins lead to food poisoning, characterized by rapid onset symptoms such as nausea, vomiting, and diarrhea, often occurring within hours of ingesting contaminated food [9]. In contrast, *E. coli* encompasses various pathotypes, notably enteropathogenic (EPEC) and enterohemorrhagic (EHEC), which utilize a range of virulent factors to mediate their effects [10]. EPEC adheres to intestinal cells via a type III secretion system that injects effector proteins into host cells, leading to cytoskeletal rearrangements and disruption of tight junctions, resulting in diarrhea [11]. EHEC produces Shiga toxin, which can cause severe complications such as hemolytic uremic syndrome [12]. Both pathogens can lead to gastrointestinal infections characterized by diarrhea and abdominal discomfort, their mechanisms ranging from toxin production in *S. aureus* to cellular invasion and immune evasion strategies in *E. coli*, highlighting the complexity of their pathogenic profiles. Multidrug-resistant strains of the isolates have raised significant concerns in food safety, especially in foodborne pathogens. Strains of *S. aureus* and *E. coli* isolated from food samples have shown resistance to multiple antibiotics, posing a challenge to public health [13].

Several studies reported that approximately 30 million people in Bangladesh suffer from foodborne illnesses each year, with acute watery diarrhea alone affecting about 0.28 million cases in 2015 [14,15]. Documented outbreak reports have linked the consumption of street-vended foods and fresh fruits to foodborne illnesses, including diarrhea, cholera, typhoid fever, and other cases of food poisoning [16].

Despite the well-documented risks, fresh-cut street-vended fruits remain a dietary staple for millions due to their affordability, convenience, and sensory appeal [17]. In the context of Bangladesh, where street vending constitutes a significant component of the urban food landscape, this study holds particular relevance [18]. For urban populations, particularly in low to middle-income settings, these fruits often represent one of the few available options for consuming fresh produce. The country’s rapid urbanization has driven a surge in demand for convenient food options, with fresh-cut fruits and vegetables emerging as a popular choice among urban dwellers [19]. A study conducted in Dhaka, Bangladesh, revealed that 48.37% of young people consume street food daily, while 15.81% consume it 2–3 times per week, highlighting a significant reliance on street food among this population [20]. However, the informal nature of street vending, coupled with limited regulatory oversight, poses significant challenges to ensuring food safety [21]. This reliance underscores the importance of understanding and mitigating the associated health risks that could disrupt food security in vulnerable communities. While numerous studies have explored the microbiological and chemical hazards in street-vended foods, there remains a notable gap in research assessing the direct health outcomes associated with their consumption [22].

Despite extensive documentation of contaminants in street-vended foods, few RCTs have directly assessed their health impacts. This study addresses that gap by evaluating the effects of consuming street-vended fresh-cut fruits in a randomized controlled trial, comparing outcomes with a control group consuming hygienically prepared foods. By combining microbial analysis of the fruits with health outcome assessment, we provide one of the first rigorous, direct evaluations of the causal links between microbial contamination and consumer health.

## 2 Materials and methods

### 2.1. Study design, setting, registration, and ethical compliance

This study employed a randomized controlled intervention design with a parallel assignment model, strictly adhering to the CONSORT (Consolidated Standards of Reporting Trials) 2025 guidelines [23]. Its primary objective was to evaluate the health impacts of consuming fresh-cut, street-vended fruits, focusing on gastrointestinal (GI) symptoms and microbial contamination. Ethical approval was obtained from the Institutional Review Board of Bangladesh Agricultural University (Approval No: AWEEC/BAU/2024(2)/15(a)). All study procedures complied with the ethical principles outlined in the 2013 amendment of the Declaration of Helsinki (1975) and the guidelines of relevant institutional and regional ethics committees governing human research [24]. The trial was registered prospectively on ClinicalTrials.gov (Identifier: NCT06858046) (https://clinicaltrials.gov/ct2/show/NCT06858046) prior to participant enrollment. This study was conducted from March 6 to March 19, 2025, in Mymensingh district, Bangladesh, an urban area where street-vended fruits are commonly consumed. Informed written consent was obtained from all adult participants prior to enrollment, following a detailed explanation of the study’s objectives, procedures, potential risks, and benefits. An impartial witness was present during each consent process to ensure participant comprehension. Participation was entirely voluntary, and individuals retained the right to withdraw at any point without penalty. Participants assigned to the control group were instructed to avoid consumption of foods that could irritate the gastrointestinal tract during the study period. All measures were taken to safeguard participant privacy by assigning unique identifiers. Health and safety protocols were strictly observed during sample collection. Confidentiality was maintained at all stages, and the collected data were used solely for research purposes.

### 2.2 Participant recruitment and group allocation

A total of 300 adults (aged 18–60) from Mymensingh district, Bangladesh, were recruited via convenience sampling. Eligible participants were willing to consume fresh-cut street-vended fruits (guava, pineapple, or watermelon) and able to provide dietary and gastrointestinal symptom data. Participants with recent use of antibiotics, antacids, or proton pump inhibitors within the last two weeks, gastrointestinal disorders, immunocompromising conditions, pregnancy/lactation, or fruit allergies were excluded. Participants were stratified by baseline gastrointestinal (GI) health (presence/absence of gastric acidity symptoms) and randomized within strata using block randomization (block size = 4) via R (set.seed = 123) to Treatment or Control groups. Allocation concealment was maintained using computer-generated randomized lists inaccessible to data collectors prior to assignment. Treatment participants were further randomized to one of three fruit types, while Control participants received no fruit (NA) with compliance monitored via phone follow-ups and food logs. Participants were not blinded due to the nature of the intervention; however, outcome assessors (survey administrators) and laboratory personnel performing microbial analyses were blinded to group allocation. Withdrawals from the Treatment group (n = 23) were replaced from a pre-randomized reserved pool (n = 80) using the same stratification and randomization procedure.

### 2.3 Sample size determination

The sample size was estimated a priori to detect a clinically meaningful 15% absolute difference in the incidence of gastrointestinal (GI) symptoms between any intervention subgroup and the control group. Based on a pilot study and previous reports showing an increase in fresh produce–associated outbreaks from 0.7% to 6% of all foodborne outbreaks, we assumed a 15% difference as both epidemiologically plausible and of public health relevance [25]. The calculation used a two-sided test with a 95% confidence level (α = 0.05) and 80% statistical power (β = 0.20). The assumed incidence of GI symptoms in the control group was 15% (*P₁* = 0.15), and in the intervention group was 30% (*P₂* = 0.30). The sample size per group (*n*) was calculated using the standard formula for comparing two independent proportions based on the z-test approximation, as described by Kim (2016) [26]:


$$n=\frac{{\left(Z_{\text{1}-\frac{\alpha }{\text{2}}}+Z_{\text{1}-\beta }\right)}^{\text{2}}\{P_{\text{1}}\left(1-P_{\text{1}}\right)+P_{\text{2}}\left(1-P_{\text{2}}\right)\}}{{\left(P_{\text{1}}-P_{\text{2}}\right)}^{\text{2}}}$$

Where $Z_{\text{1}-\frac{\alpha }{\text{2}}}=1.96$ corresponds to a 95% confidence level, $Z_{\text{1}-\beta }=0.84$ corresponds to 80% power, $P_{\text{1}}=0.15$ is the assumed incidence of GI symptoms in the control group, and $P_{\text{2}}=0.3$ is the assumed incidence in the intervention group.

Using these parameters, the minimum required sample size was approximately 118 participants per group. To accommodate stratification by gastric acidity status and fruit type (guava, pineapple, and watermelon), and to increase the statistical power for subgroup analyses, a total of 300 participants were enrolled. This included 150 participants in the control group and 150 participants distributed equally across six intervention subgroups.

### 2.4 Intervention description

Participants assigned to the treatment group were instructed to consume a single serving (~50–100g) of fresh-cut street-vended fruit, guava, pineapple, or watermelon, once, under naturalistic conditions that mimic typical public exposure. Fruits were obtained directly from licensed street vendors operating in the Mymensingh urban area during peak daytime hours (11:00 a.m. to 3:00 p.m.) to reflect real-world consumer exposure and maximize ecological validity. Vendors were randomly selected each day to minimize source bias. Each fruit was purchased fresh, cut, and packaged in a sterile zipper bag by the vendor, and then immediately delivered to the participant with minimal delay. Participants were required to consume the fruits within 20 minutes of delivery to avoid spoilage or microbial changes due to storage. Fruit allocation was randomized across acidity strata using a computer algorithm, creating six equal subgroups (e.g., Guava–Acidity, Guava–Non-Acidity) with 25 participants each. This approach allowed subgroup-specific analysis while preserving internal validity. Participants were instructed not to wash or alter the fruit prior to consumption, preserving the microbial integrity typical of street-purchased items. To monitor compliance, participants submitted timestamped photo evidence of each fruit consumption event and were contacted daily by study personnel for brief check-ins and symptom tracking. Any missed consumption or deviations were documented and analyzed separately. In contrast, participants in the control group were instructed to abstain from all street-vended fresh-cut fruits and other known gastrointestinal irritants (e.g., spicy foods, alcohol, and unpasteurized dairy) throughout the study duration. To ensure adherence, daily follow-up calls were conducted, and participants were required to report their entire dietary intake using a structured food recall template, verified during the follow-up. To ensure standardization, all study instructions (both verbal and written) were provided by trained staff. Participants received a printed dietary guide specific to their group and a fruit assignment. The intervention was designed to reflect the actual public health context of street-vended fruit consumption while upholding rigorous methodological standards required for causal inference.

### 2.5 Outcome measures and data collection procedures

The primary and secondary outcomes of this study were assessed within the first four days following the intervention. Subsequently, during a 10-day follow-up period, participants were monitored for any additional health-related complications or outcomes. For this study, the primary outcomes were defined as gastrointestinal (GI) symptoms, characterized by the occurrence of any of the following: nausea (NSA), vomiting (VMN), abdominal cramps and pain (ACP), weakness (WKS), fever (FVR), changes in stool consistency and frequency (CSCF), diarrhea (DRA), bloody diarrhea (BDRA), and heartburn (HBN).

Secondary outcomes captured multiple dimensions of gastrointestinal symptom dynamics, including the time to first occurrence of any GI symptom, symptom severity categorized as mild, moderate, or severe based on interference with daily functioning, and the duration of symptoms. During the initial four days of the study, dietary restrictions were strictly enforced to ensure compliance and minimize potential confounding exposures. This window was selected to maximize the reliability of symptom attribution to the intervention, as participants found it increasingly difficult to adhere to strict dietary controls for a prolonged duration. Beyond this period, participants were monitored daily until March 19, allowing a structured 10-day follow-up for the detection of any new or persisting GI symptoms, even though dietary adherence could not be as rigorously maintained. At baseline, each participant completed a structured questionnaire documenting their general health and confirming the absence of GI symptoms in the preceding 48 hours, which established a symptom-free reference point. Following fruit consumption, participants monitored their health daily using a structured paper-based questionnaire specifically developed for this study. The questionnaire was prepared by the World Health Organization’s Guidelines for Investigation and Control of Foodborne Disease Outbreaks and included binary yes/no entries for each symptom, as well as fields to record the time of onset and severity.

To minimize recall bias and ensure consistency, trained personnel conducted telephone interviews twice daily (morning and evening) to verify diary entries, probe for unreported symptoms, and document any medication use or dietary deviations. Automated SMS reminders were also sent to reinforce compliance. Participants in the treatment group submitted timestamped photographs as verification of fruit consumption, while control participants maintained dietary logs to confirm adherence to study restrictions. All collected forms were reviewed for completeness, digitized, and double-entered into a secure password-protected database. Discrepancies were resolved through independent cross-checking, and routine quality control audits were performed to maintain data integrity. This systematic approach ensured reliable measurement of both composite and symptom-specific outcomes, while reducing the risk of reporting or classification bias.

### 2.6. Sample collection and microbiological analysis

A 5-10g portion of each fruit sample (n = 150) intended for consumption was collected in a sterile zipper bag to determine the TVC and to isolate *S. aureus* and *E. coli*. After sample collection, the fruits were homogenized with 50 mL of peptone water using an electric homogenizer. 0.1 milliliter of the homogenized ten-fold diluted peptone water from each dilution was spread onto Plate Count Agar (HiMedia, India) and incubated at 37°C for 24 hours. Microbial load was quantified by counting colony-forming units (CFUs) according to the protocol described by Hasan et al [27]. Additionally, a portion of the peptone water was transferred to TSB (Tryptic Soy Broth) (HiMedia, India) and NB (Nutrient Broth) (HiMedia, India) for primary enrichment and then cultured on Mannitol Salt Agar (MSA) (HiMedia, India) and Eosin Methylene Blue (EMB) agar (HiMedia, India), respectively. Molecular detection of *S. aureus* targeted the *nuc* gene [28] and *E. coli* by the *16S rRNA* gene [29].

### 2.7 Statistical analysis

#### 2.7.1 Descriptive statistics and comparative analysis.

Descriptive statistics were used to summarize the frequency and percentage of each symptom in treatment and control groups, with 95% confidence intervals estimated using the Clopper–Pearson method. Group differences in symptom distribution were assessed using Pearson’s chi-square test of independence applied to 2 × 2 contingency tables, with p < 0.05 considered statistically significant. In addition, independent two-sample t-tests assuming unequal variance were performed to compare mean binary symptom values between groups. Relative risks (RR) with 95% confidence intervals were also calculated to quantify the magnitude of treatment effects, providing both statistical and clinical insights into the strength of associations.

#### 2.7.2 Survival and cox proportional hazards analysis.

Kaplan–Meier survival analysis and Cox proportional hazards modeling were conducted to evaluate the relationship between fruit consumption, group assignment, and gastrointestinal symptom onset. Symptoms were binarized (Yes = 1, No = 0) to define the event variable, while time-to-onset (TON) was used as the duration, with censoring at 96 hours for participants without symptoms. Kaplan–Meier survival curves were generated for all subjects and stratified by group, with 95% confidence intervals. For multivariable analysis, predictors (Group, Fruit, Group Acidity) and confounders (Age, Sex, Hygienic Practice Score) were included in a Cox proportional hazards model, with categorical variables converted to dummy variables. Model assumptions were checked, and multicollinearity was assessed using variance inflation factors (VIF).

#### 2.7.3 Correlation, regression, and FDR correction.

To evaluate associations between microbial contamination (*S. aureus*, *E. coli*, and log-transformed TVC) and gastrointestinal symptoms, multiple statistical approaches were implemented in Python. Binary variables were encoded numerically, and correlations were assessed using Spearman’s rank, Pearson’s, and point-biserial coefficients. Logistic regression models were fitted to estimate odds ratios (ORs) with 95% confidence intervals, and Mann-Whitney U tests were conducted to compare microbial loads between symptomatic and asymptomatic groups. P-values were corrected using the Benjamini-Hochberg False Discovery Rate (FDR) method (q < 0.10). Dose–response relationships between log_TVC and symptom probabilities were visualized using logistic regression, with predicted probabilities.

#### 2.7.4 Receiver operating characteristic (ROC) curve analysis.

The study analyzed symptom-based predictors of bacterial contamination using both intention-to-treat (ITT) and per-protocol (PP) populations, where ITT included all participants from the treatment group and PP was restricted to individuals with complete records. Symptom variables (NSA, VMN, ACP, WKS, FVR, DRA, BDRA, CSCF, HBN) and bacterial detections (*S. aureus* and *E. coli*) were dichotomized, while TVC were log₁₀-transformed to reduce skewness. Receiver operating characteristic (ROC) curve analyses were performed to assess the discriminatory ability of each symptom against bacterial loads, with the area under the curve (AUC) and corresponding 95% confidence intervals estimated using the Hanley and McNeil method. The Youden Index was calculated to determine optimal thresholds along with sensitivity and specificity. In parallel, log-rank tests were applied to evaluate the association of symptom occurrence with bacterial load, stratified by median values for TVC or positivity for *S. aureus* and *E. coli*. For each bacterial load, the top three symptoms were identified based on AUC values, and results were summarized in tabular and graphical formats, including six-panel ROC plots and detailed reports of AUC metrics, optimal cut-offs, Youden Index values, and log-rank statistics for both ITT and PP analyses. All the statistical analysis was performed using several libraries and packages of Python (v.3.13.7) and R (v.4.5.1).

## 3 Results

### 3.1 Participant flow

A total of 639 individuals were assessed for eligibility. Of these, 259 participants were excluded based on predefined exclusion criteria. The remaining 300 eligible participants were enrolled in the study and randomized into two groups: 150 assigned to the Treatment group and 150 assigned to the Control group. In addition to the enrolled participants, 80 pre-screened eligible individuals were assigned to a reserved group to account for potential dropouts and ensure continuity in intervention and data integrity. Randomization and fruit assignment procedures were applied to these reserved participants following the same protocol used for the main study cohort. During the intervention period, 23 participants in the Treatment group withdrew from the study. The attrition included 12 participants from the Guava group, 5 from the Pineapple group, and 6 from the Watermelon group. To maintain group balance and statistical power, 23 participants were selected from the reserved group, all of whom had previously been randomized to the Treatment arm and assigned fruit types using stratified block randomization. These participants were integrated into the ongoing study without disrupting the allocation ratio or fruit group distribution. Thus, the final ongoing sample consisted of 150 participants in the Treatment group (127 original + 23 replacements) and 150 participants in the Control group, all of whom continued through the intervention phase. A complete visualization of the participant flow is presented in (S1 Fig).

### 3.2 Baseline characteristics

The study included 300 participants, evenly divided between the Control (n = 150) and Treatment (n = 150) groups. Participants were aged between 18 and 60 years, with mean ages of 28.63 years (SD = 8.00) in the Control group and 28.56 years (SD = 8.28) in the Treatment group. Sex distribution was balanced, with females representing 46% and males 54% in the Control group, compared to 50% for both sexes in the Treatment group. The majority of participants were aged between 21–30 years (Control: 79; Treatment: 84), followed by those over 30 (Control: 61; Treatment: 56), and a smaller proportion under 21 (10 in each group). Fruit consumption was recorded only for the Treatment group, with an overall mean intake of 74.57 grams (SD = 14.6), ranging from 50 to 100 grams. When broken down by fruit type, participants consumed an average of 75.94 grams (SD = 14.64) of guava, 72.98 grams (SD = 14.13) of pineapple, and 74.79 grams (SD = 15.16) of watermelon. These amounts were measured across 50 participants per fruit type. The comparable demographic characteristics and balanced distributions between groups in (S1 Table) provide a robust foundation for subsequent comparisons and analyses.

### 3.3 Health symptoms after consumption of fruits

#### 3.3.1 Primary outcomes after fruit consumption.

**Fig 1:**
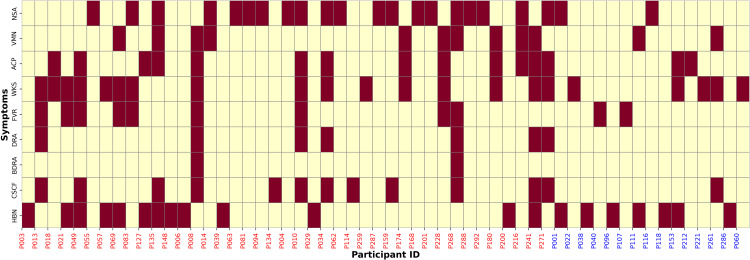
Heatmap of gastrointestinal symptoms by participant- X-axis: Participant IDs (Red  = Treatment Group [P003–P271], Blue = Control Group [P001–P060]); Y-axis: Reported Symptoms.

**Fig 2:**
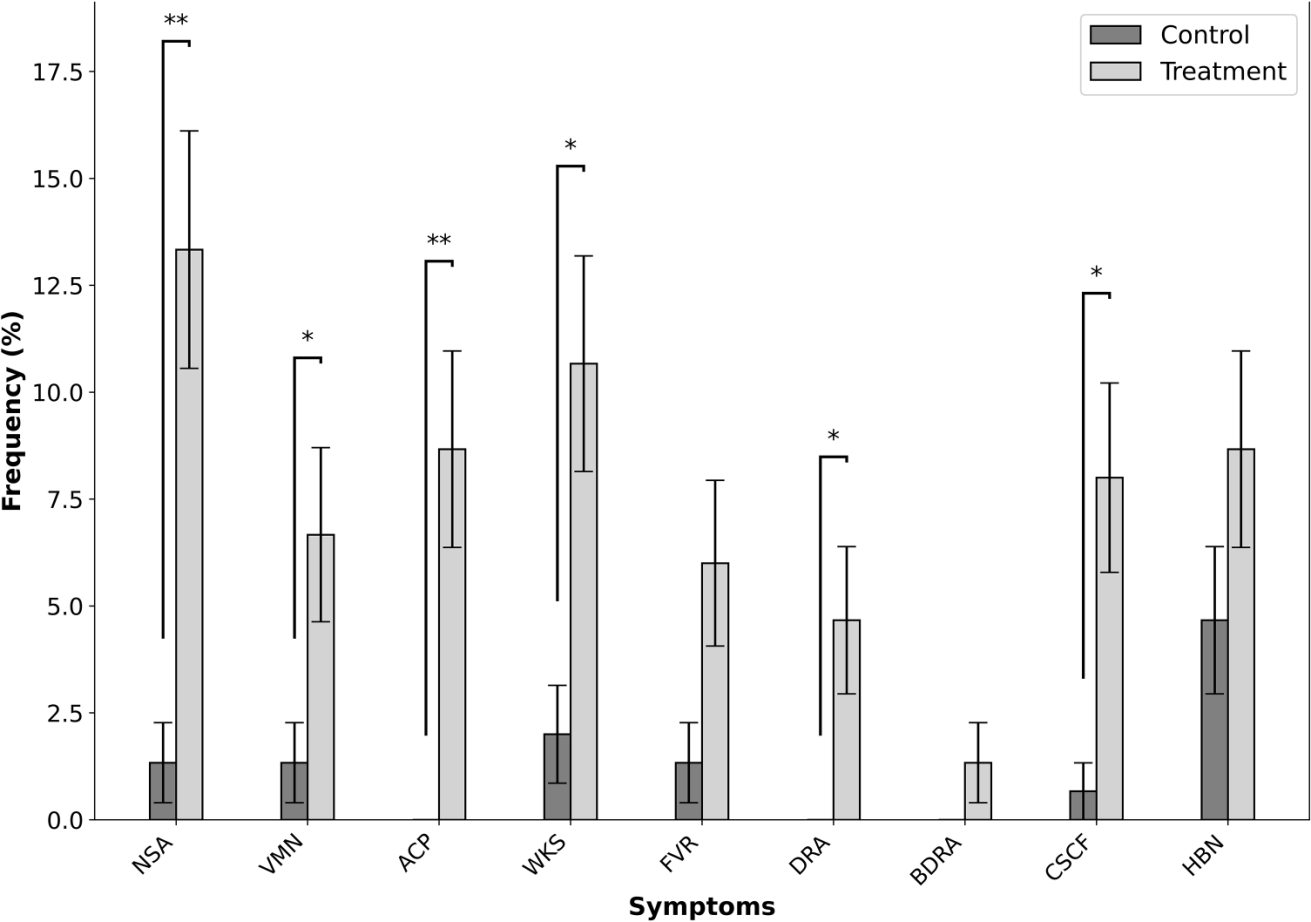
Showing the frequency of different symptoms in the control and treatment groups, with significant differences indicated by asterisks (*p < 0.05, **p < 0.001).

The analysis of symptom prevalence and onset revealed significant differences between the treatment and control groups, painting a compelling picture of the potential impact of exposure. A total of 41 participants (27.3%) from the treatment group (n = 150) and 15 participants (10%) from the control group (n = 150) experienced at least one symptom during the observation period (Fig 1) and (S2 Table). From the symptoms, NSA emerged as a stark indicator, affecting 13.33% of participants in the treatment group compared to only 1.33% in the control group. This translated to a relative risk (RR) of 10 (95% CI: 2.38–42.03, *p* < 0.001), underscoring a robust association (Fig 2). Similarly, VMN was observed in 6.67% of the treatment group versus 1.33% in the control group, with an RR of 5 (95% CI: 1.11–22.44, *p* = 0.039). ACP, a symptom exclusive to the treatment group, were reported by 8.67% of participants (*p* < 0.001), highlighting a significant relationship. Further insights emerged as WKS was reported by 10.67% of individuals in the treatment group compared to 2% in the control group, with an RR of 5.33 (95% CI: 1.59–17.92, *p* = 0.004). FVR, while more frequent in the treatment group (6%) than in the control group (1.33%), demonstrated a marginally non-significant association (*p* = 0.065). DRA was reported in 4.67% of the treatment group, while no cases were found in the control group (*p* = 0.02). Although BDRA was rare, affecting 1.33% of the treatment group and none in the control group, the association did not reach statistical significance (*p* = 0.48). CSCF presented a particularly striking difference, affecting 8% of the treatment group compared to only 0.67% in the control group, with an RR of 12 (95% CI: 1.58–91.14, *p* = 0.004). HBN, while present in 8.67% of the treatment group and 4.67% of the control group, showed no significant association (*p* = 0.2).

Logistic regression analysis identified hygienic practices as a significant factor influencing gastrointestinal symptom risk in the Control group. Higher hygienic practice scores were associated with a reduced likelihood of developing VMN (OR = 0.81, p = 0.014), ACP (OR = 0.83, p = 0.024), WKS (OR = 0.75, p < 0.001), and FVR (OR = 0.73, p = 0.010), indicating a protective effect. In contrast, no statistically significant associations were observed in the Treatment group, suggesting that high exposure or other environmental factors may have overridden the protective effect of hygiene, making group assignment (Treatment vs. Control) a key determinant of increased gastrointestinal symptom risk.

#### 3.3.2 Secondary outcomes after consumption of fruits.

**Fig 3:**
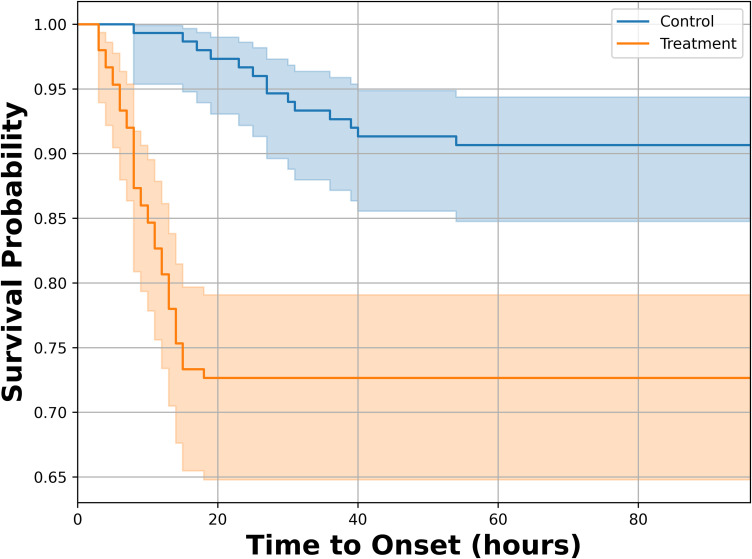
Showing the Kaplan-Meier survival curves of the treatment and control group for the symptoms in relation to time to onset (Hours).

As secondary outcomes of the study, the stratified analysis of participants revealed variations in illness duration and time to onset (TON) across severity levels and group acidity. The comparison of these metrics for the Treatment group across different severity strata is summarized in (S2 Fig). Furthermore, the difference in the time to symptom onset between the Control and Treatment groups was analyzed using a survival probability curve, as shown in Fig 3. For severity, participants with mild symptoms in the Treatment group had a mean duration of 3.7 ± 0.44 hrs and a mean TON of 9.25 ± 0.92 hrs, whereas the Control group had slightly longer illness duration (4.5 ± 0.90 hrs) and substantially higher TON (28.62 ± 3.34 hrs). Moderate cases in the Treatment group showed a mean duration of 14.62 ± 2.15 hrs and TON of 10.15 ± 1.14 hrs, compared with the Control group’s duration of 17 hrs and TON of 19 hrs. Severe cases in the Treatment group had markedly longer illness duration (49.63 ± 6.48 hrs) but similar TON (9.5 ± 1.20 hrs), while no events were observed in the Control group. When stratified by group acidity, participants in the HAP (Having Acidity Problems) category of the Treatment group had a mean duration of 15.77 ± 3.89 hrs and TON of 9.27 ± 0.72 hrs, whereas the Control group had a shorter duration (5.75 ± 1.65 hrs) but higher TON (26.6 ± 2.50 hrs). In the ANAP (Apparently No Acidity Problems) category, the Treatment group exhibited a mean duration of 16.73 ± 5.06 hrs and TON of 10.13 ± 1.12 hrs, compared with the Control group’s duration of 4.5 ± 1.32 hrs and TON of 31.25 ± 10.01 hrs. Overall, the Treatment group consistently demonstrated lower time to onset across both severity and acidity strata, while illness duration was longer in moderate to severe cases.

The Cox proportional hazards model further examined the association of fruit consumption, group assignment, acidity, age, sex, and hygienic practice score with the time to symptom onset. Treatment group participants had a markedly higher hazard of developing symptoms (HR = 162.68, 95% CI: 21.53–1229.43, p < 0.001), while higher hygienic practice scores were associated with a modest but significant reduction in hazard (HR = 0.90, 95% CI: 0.82–0.99, p = 0.028). Other covariates, including age (HR = 0.98), fruit type (pineapple HR = 0.51, watermelon HR = 0.56), acidity problem status (HAP HR = 1.45), and sex (male HR = 0.90), were not statistically significant. Multicollinearity was minimal, as indicated by variance inflation factors (VIFs < 2.2 for all predictors, except the constant). The Schoenfeld residuals test suggested that the proportional hazards assumption was generally met (all p > 0.05, except for the HAP group, p = 0.033), indicating the effects of most covariates were stable over time. Overall, the model demonstrated good predictive performance with a concordance index of 0.76 and showed an acceptable fit with an AIC of 302.13, supporting its reliability in assessing risk factors for symptom development in this population.

#### 3.3.3 Primary outcomes in the treatment group based on GI acidity problems.

**Fig 4:**
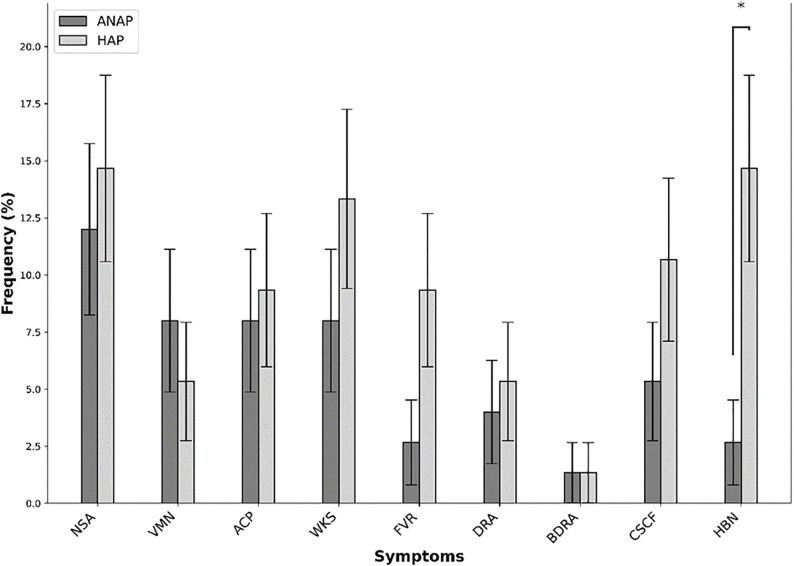
Frequency of symptom occurrences in groups with and without a history of acidity problems, with significant differences indicated by asterisks (*p < 0.05, **p < 0.001).

The comparative analysis between the HAP and ANAP groups revealed a notable difference in the prevalence of HBN, which was significantly higher in the HAP group (14.67%) compared to the ANAP group (2.67%) (*p* = 0.009), with a relative risk (RR) of 5.5 (95% CI: 1.26–23.98), indicating that individuals in the HAP group were over five times more likely to experience heartburn (Fig 4). Although other symptoms such as NSA, VMN, ACP, WKS, FVR, DRA, and CSCF varied in distribution between groups, none of these differences reached statistical significance.

#### 3.3.4 Primary outcomes in the groups based on fruits.

The prevalence of symptoms varied across different fruit groups. In the Guava group, the most prevalent symptoms were NSA, with a prevalence of 18.0% (95% CI: 8.0%, 30.0%), followed by WKS and HBN, both at 16.0% (95% CI: 6.0%, 26.0%). For the Pineapple group, NSA was again the most prevalent symptom at 18.0% (95% CI: 8.0%, 30.0%), followed by VMN at 8.0% (95% CI: 2.0%, 16.0%). In the Watermelon group, NSA was the most prevalent symptom at 16.0% (95% CI: 6.0%, 26.0%), followed by ACP at 12.0% (95% CI: 4.0%, 22.0%). These results show that NSA was consistently prominent across all fruit groups, while other symptoms varied.

### 3.4 Microbiological analysis

#### 3.4.1 Bacterial load and correlation analysis with GI symptoms.

The TVC (CFU/ml) measured in 50 samples of guava showed a mean log TVC of 5.86 ± 0.75, while 50 pineapples and 50 watermelons had mean log TVCs of 5.78 ± 0.63 and 5.79 ± 0.73, respectively (S3 and S4 Figs). The correlation analysis between bacterial load and gastrointestinal (GI) symptoms revealed significant associations for several symptoms, emphasizing the impact of microbial contamination on symptom manifestation (S3 Table).

WKS exhibited the strongest association with bacterial load, followed by ACP and DRA, all of which showed strong correlations according to Cohen’s guidelines (r ≥ 0.5). The point-biserial correlation coefficients were r = 0.66 for WKS, r = 0.56 for ACP, and r = 0.51 for DRA (all p < 0.001). Logistic regression analyses further confirmed these findings, yielding odds ratios (OR) of 11.28 (95% CI: 4.48–28.43, p < 0.001) for WKS, 6.16 (95% CI: 2.87–13.23, p < 0.001) for ACP, and 6.73 (95% CI: 2.56–17.70, p < 0.001) for DRA, reflecting substantially increased risks at higher bacterial counts. Mann-Whitney U tests also supported these results (U = 1588 for ACP and U = 715 for DRA, both p < 0.001), and the associations remained significant after adjustment for multiple comparisons (adjusted p < 0.001 and 0.010, respectively).

Other symptoms, including FVR and CSCF, also showed moderate positive correlations with bacterial load, with r values of 0.53 and 0.49, respectively (both p < 0.001). Logistic regression revealed elevated odds ratios for these symptoms as well-6.19 (95% CI: 2.64–14.54, p < 0.001) for FVR and 4.95 (95% CI: 2.40–10.21, p < 0.001) for CSCF-indicating a dose-dependent increase in symptom risk with rising bacterial load. Corresponding Mann-Whitney U tests were highly significant (all p < 0.005), and these associations withstood adjustment for multiple testing.

NSA and Heartburn HBN exhibited weaker correlations with bacterial load. NSA showed r = 0.26 (p = 0.001), OR = 2.16 (95% CI: 1.29–3.65, p = 0.004), and Mann-Whitney U = 2057 (p = 0.027). HBN demonstrated r = 0.19 (p = 0.020) and OR = 1.94 (95% CI: 1.06–3.54, p = 0.031), although these associations did not consistently reach significance after multiple testing correction (adjusted p = 0.056). In contrast, BDRA showed no meaningful association with bacterial load (r = 0.06, p = 0.49; OR = 1.62, p = 0.50), which was further supported by a non-significant Mann-Whitney U test (p = 0.95).

**Fig 5:**
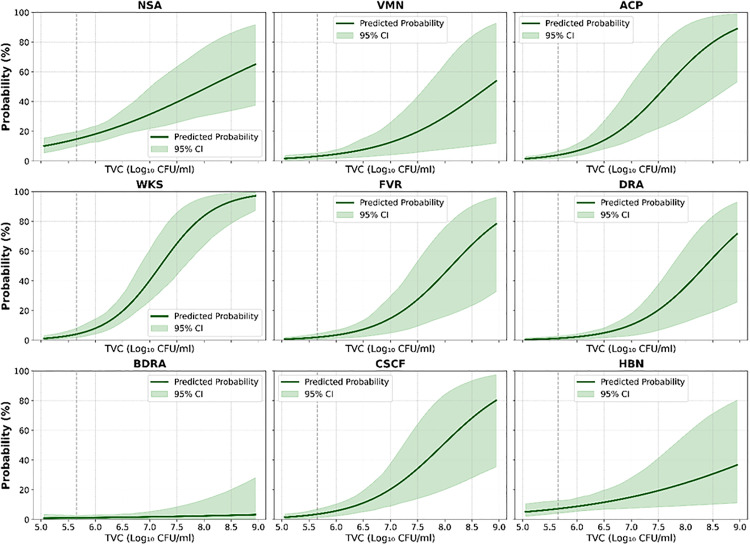
Dose-response curve illustrating the relationship between Total Viable Count (TVC) (log scale) and various symptoms.

The dose-response curves illustrated these relationships by demonstrating a clear, dose-dependent increase in the probability of symptom manifestation with rising bacterial counts (Fig 5). For diarrhea, at a mean TVC of 7.57 ± 1.41 log CFU/ml, the predicted probability exceeded 80% when TVC levels were above 7.5 log CFU/ml. Fever showed a similar trend, with a mean TVC of 7.36 ± 1.15 log CFU/ml and predicted symptom probability surpassing 70% at higher bacterial loads. Nausea’s probability curve, however, remained relatively flat across the bacterial load range, consistent with its weaker correlation. Weakness and abdominal cramps followed intermediate trajectories, with steadily rising symptom probabilities as TVC increased, whereas CSCF showed a more gradual increase, requiring higher bacterial loads to reach notable symptom risk.

#### 3.4.2 Prevalence of *S. aureus* and *E. coli* and correlation with symptoms.

*S. aureus* and *E. coli* were identified using cultural, staining, biochemical, and molecular methods. For *S. aureus*, colonies exhibited distinct golden-yellow pigmentation on Mannitol Salt Agar (MSA) (S5 Fig). Gram staining revealed *S. aureus* as Gram-positive cocci in grape-like clusters (S6 Fig). Biochemical tests confirmed its identity, showing positive results in the catalase and coagulase tests, while the oxidase test was negative. Molecular confirmation was achieved by detecting the *nuc* gene through PCR (Lane L- 100 bp Ladder; Lane 1–3 DNA at 279 bp; 4- Negative Control; 5 Positive Control) (S7 Fig). For *E. coli*, colonies displayed a characteristic metallic green sheen on Eosin Methylene Blue (EMB) agar (S8 Fig). Gram staining identified *E. coli* as Gram-negative, short rod-shaped bacteria (S9 Fig). Biochemical tests further confirmed *E. coli* through a positive indole test, a positive methyl red (MR) test, and negative results in both Voges-Proskauer (VP) and citrate utilization tests. Molecular identification was confirmed by detecting the *16S rRNA* gene via PCR (Lane L- 100 bp Ladder; Lane 1–3 DNA at 279 bp; 4- Positive Control; 5- Negative Control) (S10 Fig). These combined approaches ensured the accurate identification of both bacterial species.

**Fig 6:**
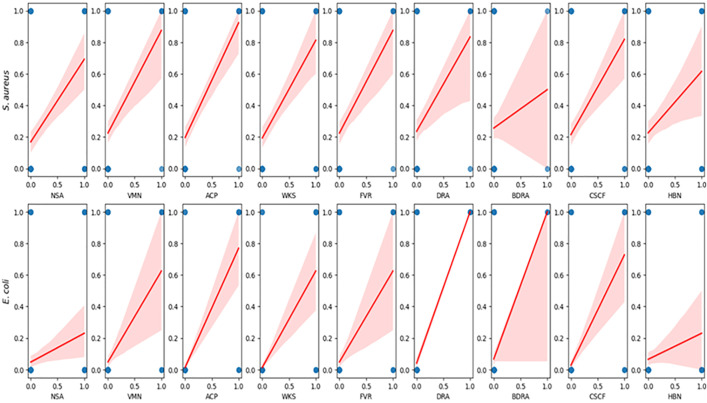
Correlation between the presence of S. aureus and E. coli with gastrointestinal symptoms, identified by Spearman correlation analysis. In the figure, 0 and 1 on the x-axis denote symptom absence or presence, while 0 and 1 on the y-axis indicate the presence of S. aureus (top row) and E. coli (bottom row), with positive correlations suggesting higher symptom occurrence with bacterial presence.

Among the fruits tested for bacterial contamination, guava showed the highest prevalence of *S. aureus* at 34%, while pineapple had the lowest prevalence at 20% (Table 1). For *E. coli*, the highest prevalence was observed in watermelon at 10%, and the lowest in pineapple at 6%. Spearman’s correlation analysis as illustrated in Fig 6 revealed significant associations between bacterial prevalence and gastrointestinal symptoms. For *S. aureus*, moderate positive correlations were observed with NSA (ρ = 0.45, *p* < 0.001), ACP (ρ = 0.47, *p* < 0.001), WKS (ρ = 0.44, *p* < 0.001), and FVR (ρ = 0.33, *p* < 0.001), indicating consistent symptom linkage. *E. coli* demonstrated even stronger correlations with ACP (ρ = 0.78, *p* < 0.001), WKS (ρ = 0.69, **p* *< 0.001), DRA (ρ = 0.69, *p* < 0.001), and CSCF (ρ = 0.67, *p* < 0.001). Additional significant associations included (VMN, ρ = 0.48, *p* < 0.001) and (NSA, ρ = 0.25, *p* = 0.002). In contrast, BDRA showed a weak and non-significant correlation with *S. aureus* (ρ = 0.06, *p* = 0.439), while a moderate and significant correlation was observed with *E. coli* (ρ = 0.39, *p* < 0.001). Complementary Pearson correlation analysis yielded consistent results (S4 Table), with strong positive relationships between *E. coli* and ACP (r = 0.78), WKS (r = 0.69), DRA (r = 0.69), and CSCF (r = 0.67), as well as between *S. aureus* and NSA (r = 0.45), ACP (r = 0.47), and WKS (r = 0.44). These findings collectively reinforce the association between bacterial contamination and the onset of gastrointestinal and systemic symptoms.

**Table 1:** Prevalence of *S. aureus* and *E. coli* isolated from guava, pineapple, and watermelon.

Fruit	Bacteria	Prevalence (%)	Lower CI (%)	Upper CI (%)
Guava	*S. aureus*	34	22.4	47.8
	*E. coli*	8	3.2	18.8
Pineapple	*S. aureus*	20	11.2	33
	*E. coli*	6	2.1	16.2
Watermelon	*S. aureus*	24	14.3	37.4
	*E. coli*	10	4.3	21.4

### 3.5 ROC analysis for TVC and microbial contamination with the symptoms

**Fig 7:**
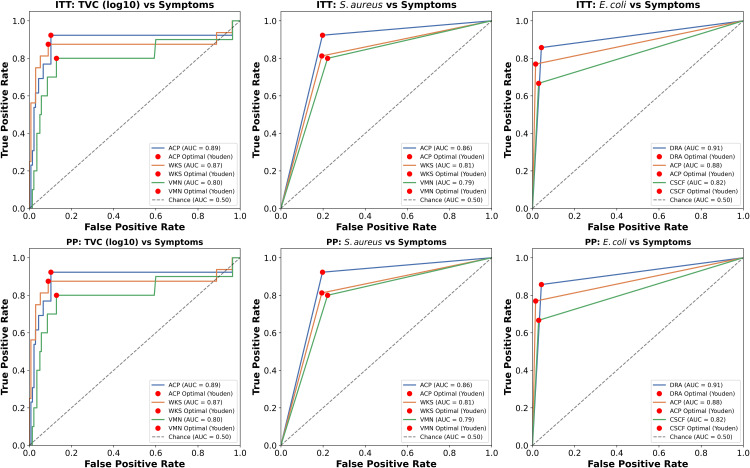
ROC curves of TVC and *S. aureus, E. coli* with symptoms.

In the ITT and PP populations (n = 150 each), ACP, WKS, and VMN in (Fig 7) were the most strongly associated symptoms with TVC (log₁₀), with ACP showing AUC = 0.892 (95% CI: 0.774–1.009), Youden Index = 0.821, optimal threshold = 5.993, sensitivity = 0.923, specificity = 0.898, and log-rank χ² = 10.123 (p = 0.001); WKS had AUC = 0.868, Youden Index = 0.785, threshold = 5.993, sensitivity = 0.875, specificity = 0.910, χ² = 10.007 (p = 0.002); VMN showed AUC = 0.801, Youden Index = 0.671, threshold = 5.993, sensitivity = 0.800, specificity = 0.871, χ² = 3.831 (p = 0.050). For *S. aureus*, ACP, WKS, and VMN had AUCs of 0.863, 0.809, and 0.789 with Youden Indices of 0.726, 0.618, and 0.579, thresholds = 1.0, sensitivities 0.923–0.800, specificities 0.803–0.806, and χ² 32.310–16.130 (all p ≤ 0.001). For *E. coli*, DRA, ACP, and CSCF were top, with DRA showing AUC = 0.908, Youden Index = 0.815, threshold = 1.0, sensitivity = 0.857, specificity = 0.958, χ² = 59.851 (p < 0.001), ACP AUC = 0.877, Youden Index = 0.755, sensitivity = 0.769, specificity = 0.985, χ² = 91.256, and CSCF AUC = 0.819, Youden Index = 0.638, sensitivity = 0.667, specificity = 0.971, χ² = 60.589. Similar patterns were observed in the PP population, indicating robust associations across analyses.

## 4 Discussion

The findings of this study highlight critical health risks associated with consuming street-vended fruits, underscoring the interplay between bacterial contamination and symptom manifestation. Gastrointestinal symptoms such as nausea, vomiting, abdominal cramps and pain, and diarrhea, were markedly more prevalent in the treatment group, reflecting the broader impact of microbial exposure through contaminated food sources. These symptoms appeared more frequently and progressed more rapidly among individuals exposed to contaminated fruits, suggesting a swift activation of gastrointestinal responses to potential pathogens. This aligns with findings from previous studies emphasizing the urgency of addressing food hygiene practices to mitigate acute health impacts, particularly among vulnerable populations [30–32].

However, participants from the control group also experienced some gastrointestinal symptoms, despite not consuming the fruits provided to the treatment group. These symptoms could potentially be attributed to underlying pathophysiological conditions of the gastrointestinal tract (GIT), lifestyle factors, and hygienic practices [33].

One of the most significant findings was the ten times increase in the relative risk of nausea in the treatment group compared to the control group, with a highly significant p-value, making nausea a key indicator of foodborne gastrointestinal distress. This observation resonates with the findings reported by Horn C. [34]. Abdominal cramps and pain, absent in the control group but reported by 8.67% of the treatment group, emerged as another critical symptom. This could be attributed to the production of bacterial toxins or the invasion of the intestinal lining, leading to inflammation and irritation, as described by Martín-Rodríguez et al [35]. Although diarrhea was less frequent, its strong correlation with bacterial contamination levels highlights its role as a marker of severe microbial infection. These findings aligned with previous studies emphasizing the acute health impacts of contaminated foods, underlining the urgent need for preventive measures [36,37].

Group-specific analyses further highlighted heightened vulnerability among individuals with pre-existing acidity problems, who experienced a significantly higher prevalence of heartburn, suggesting that underlying gastrointestinal conditions may exacerbate localized symptoms. This might be due to the use of spices with fresh-cut fruits, which is aligned with the findings of Surdea-Blaga et al, [38]. However, the differences are not statistically significant. The prevalence of symptoms varied across different fruit groups, with nausea being the most common symptom in guava, pineapple, and watermelon, while other symptoms showed a nonsignificant variation within each group.

Temporal analyses revealed notable differences in illness progression between the Treatment and Control groups. Stratified analysis showed that the Treatment group consistently experienced earlier symptom onset across both severity levels and participants with acidity-related conditions, suggesting accelerated symptom manifestation, while illness duration tended to be longer in moderate to severe cases. The Cox proportional hazards analysis further indicated that being in the Treatment group was a primary driver of increased symptom risk, whereas individual characteristics such as age and sex did not significantly influence onset. Conversely, better hygienic practices appeared to modestly reduce the hazard of symptom development, highlighting the protective role of hygiene under lower exposure conditions. Overall, these findings suggest that early onset in the Treatment group may reflect heightened susceptibility or interactions with environmental and exposure factors, consistent with prior reports of post-ingestion gastrointestinal infections and enteropathogenic dynamics [9,39,40].

The bacterial load assessment provided an average of 1.385 × 10^7^ CFU/ml per sample, which is aligned with several studies conducted in Bangladesh [6,41]. The higher bacterial load observed in symptomatic individuals may reflect inadequate hygiene during food handling, storage, or preparation, even though vendor practices were not directly assessed in this study. A study in Bangladesh reported that street-vended foods are often exposed to multiple points of contamination, including improper washing, poor water quality, and cross-contamination during cutting and serving [42]. Such conditions provide favorable opportunities for bacterial growth and toxin production, ultimately raising the risk of gastrointestinal illness. The findings therefore emphasize the importance of enforcing proper hygiene and food safety practices at every stage of the food chain, particularly in informal or unregulated vending environments.

The microbiological evidence obtained in this study provides strong support for the association between bacterial contamination and gastrointestinal symptom severity. Severe symptoms like diarrhea, abdominal pain, and weakness were closely linked with elevated TVC, underscoring the dose-dependent relationship between microbial burden and clinical outcomes. Similar associations between TVC and foodborne illness have been reported in several studies [16,43], which strengthens the external validity of these findings. This alignment with existing literature highlights the value of using TVC as a predictive marker for food safety and public health monitoring.

From a biological perspective, the results suggest that both bacterial load and toxin production contribute to the observed symptom patterns. At higher contamination levels, bacteria not only colonize the gastrointestinal tract more effectively but also release greater quantities of enterotoxins and cytotoxins [44]. These toxins can disrupt intestinal epithelial integrity, trigger inflammation, and disturb fluid and electrolyte balance, leading to severe manifestations such as diarrhea, abdominal cramps, and systemic symptoms like fever [45]. By contrast, milder symptoms such as nausea or heartburn appear less strongly related to bacterial dose, reflecting a lower threshold of irritation or toxin sensitivity.

This study found a notable prevalence of *S. aureus* and *E coli* in fresh-cut guava, pineapple, and watermelon. The sources of contamination are likely attributed to improper handling practices and the use of contaminated water during fruit processing [46,47]. Spearman correlation analyses implicated *S. aureus* and *E. coli* as primary contributors***. S. aureus*** was associated with symptoms such as nausea, abdominal cramps, and pain, likely due to its production of enterotoxins such as SEA, SEB, and SEC, which act as superantigens and stimulate an exaggerated immune response [48]. Meanwhile, ***E. coli*** showed strong associations with diarrhea and systemic symptoms, which might be due to its production of Shiga toxin and enterotoxins, disruption of epithelial barrier integrity, and induction of inflammation, consistent with its well-documented role in gastrointestinal pathogenesis [49].

ROC analysis demonstrated the strong diagnostic value of microbial indicators in predicting gastrointestinal symptoms. The high discriminatory power of specific pathogens and bacterial load supports their use as reliable markers for foodborne illness risk. These findings advocate for integrating microbial profiling into routine food safety surveillance, especially in high-risk environments like street food vending where rapid, evidence-based interventions are vital.

These findings underscore the need for targeted policy and regulatory measures to ensure food safety in informal markets. Structured vendor training, improved hygiene practices, and strict enforcement of safety guidelines, combined with consumer education on safe food handling, can reduce bacterial contamination and lower the risk of gastrointestinal illness. Future research should explore host-pathogen interactions, delayed symptom onset, and environmental factors influencing contamination to inform effective public health strategies.

## 5 Strengths and limitations

This study employed a randomized controlled trial design with stratified randomization and adherence to CONSORT 2025 guidelines, ensuring methodological rigor. Microbiological assessments were conducted in a blinded manner, and participant compliance was closely monitored using daily photo verification and structured follow-ups. Multiple statistical methods, including survival analysis and correlation modeling, enabled a comprehensive evaluation of the relationship between fruit consumption and gastrointestinal outcomes. Nonetheless, several important limitations should be acknowledged. The non-blinded design may have introduced reporting or placebo effects, while self-reported symptoms remain susceptible to recall bias. Dietary variations outside the intervention, such as consumption of other foods or beverages, were not fully controlled, potentially confounding symptom associations. Seasonal and environmental factors, including weather and fruit type variability, may have influenced microbial contamination and symptom onset. Additionally, while microbiological testing was performed for total viable count, *S. aureus*, and *E. coli*, other pathogens were not assessed, limiting causal inference. Differences in healthcare-seeking behavior, the relatively small control group, the short follow-up period, and the urban study setting may further constrain the generalizability of findings. These limitations highlight the need for future studies to incorporate objective symptom monitoring, expanded pathogen testing, and detailed dietary and environmental data.

## 6 Conclusion

The study demonstrates that consumption of street-vended fresh-cut fruits is significantly associated with gastrointestinal symptoms, particularly nausea, vomiting, abdominal cramps, weakness, and diarrhea. Participants in the Treatment group exhibited earlier symptom onset and higher symptom prevalence compared to controls, with bacterial contamination, especially *E. coli* and *S. aureus* emerging as key drivers of risk. Temporal, microbiological, and statistical analyses collectively indicate a clear dose-response relationship between microbial load and symptom manifestation, underscoring the importance of food safety, education, hygiene practices, and microbial monitoring to prevent foodborne illness from fresh-cut fruits.

## Supporting information

S1 Fig Flowchart of participant recruitment, eligibility assessment, enrollment, and group allocation in the study. (TIFF)

S2 Fig Mean time to symptom onset, severity, and duration of illness of the participant groups. (TIFF)

S3 Fig Bacterial colonies cultured on Plate Count Agar (PCA) from a 10 ⁻ ⁶ dilution. (TIFF)

S4 Fig Bacterial load (CFU/ml) in guava, pineapple, and watermelon was identified by TVC. (TIFF)

S5 Fig Growth of Staphylococcus aureus on Mannitol Salt Agar (MSA), showing characteristic yellow colonies. (TIFF)

S6 Fig Staining morphology of Staphylococcus aureus, revealing grape-like clusters of cocci under microscopy. (TIFF)

S7 Fig Amplification of 279 bp fragment of *nuc* gene of *S. aureus* by PCR. Lane L: 100 bp size DNA marker; Lane 1–3: DNA samples *of S. aureus* extracted from guava, pineapple, and watermelon; Lane 4: Negative control; Lane 5: Positive control.
(TIFF)

S8 Fig Growth of *Escherichia coli* on Eosin Methylene Blue (EMB) Agar, showing characteristic metallic green sheen. (TIFF)

S9 Fig Staining morphology of Escherichia coli, showing characteristic rod-shaped cells under microscopy. (TIFF)

S10 Fig Amplification of 585 bp fragment of *16S RNA* gene of *E. coli* by PCR. Lane L: 100 bp size DNA marker; Lane 1–3: DNA samples *of E. coli* extracted from guava, pineapple, and watermelon; Lane 3: Positive control. Lane 5: negative control.
(TIFF)

S1 Table Baseline demographics and hygienic scores among the participants. (DOCX)

S2 Table Frequency of symptoms among the participants. (DOCX)

S3 Table Association between bacterial load (log_TVC) in the fruit samples and the Gastrointestinal Symptoms. (DOCX)

S4 Table Spearman and Pearson correlation between S. aureus and E. coli and GI symptoms after consumption of fruits. (DOCX)
