# Supplementary figures and images for: Health effects of street vended fresh cut fruits: A randomized controlled trial in Bangladesh
Source: PLoS One. 2025 Oct 31;20(10):e0335979. doi: 10.1371/journal.pone.0335979 (PMC12578160; doi:10.1371/journal.pone.0335979)

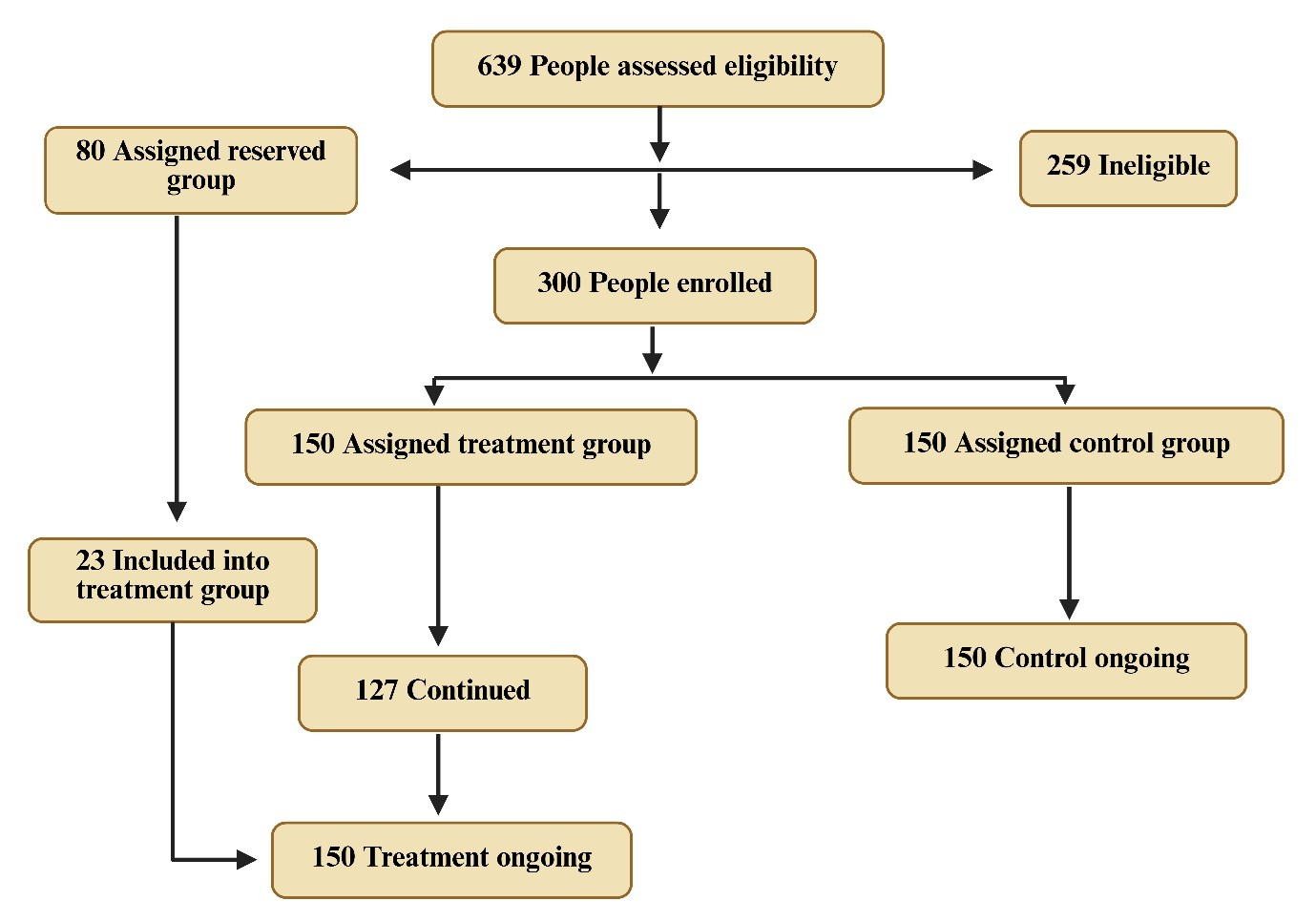

Supplement: S1 Fig — (TIFF) [file pone.0335979.s001.tiff]

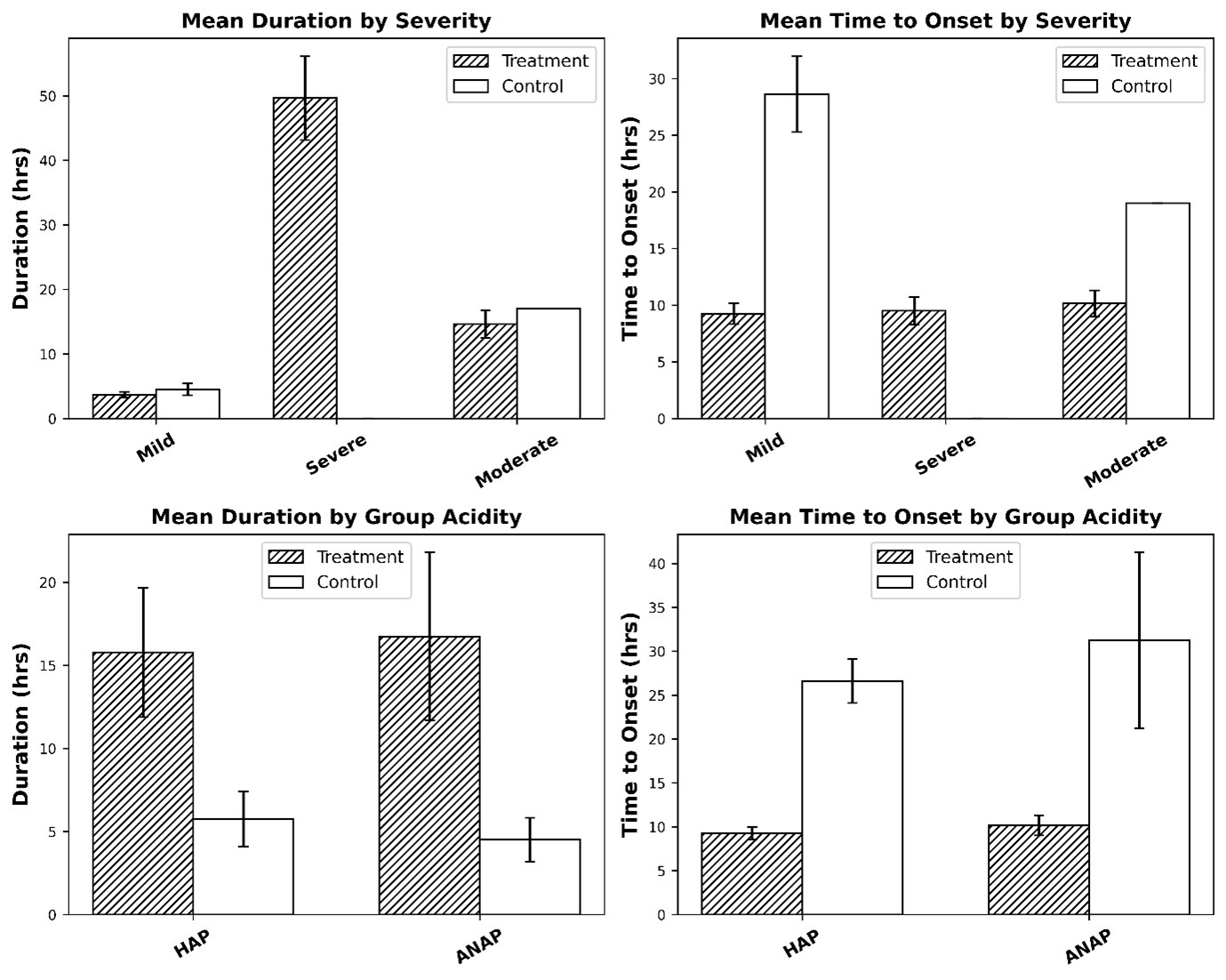

Supplement: S2 Fig — (TIFF) [file pone.0335979.s002.tiff]

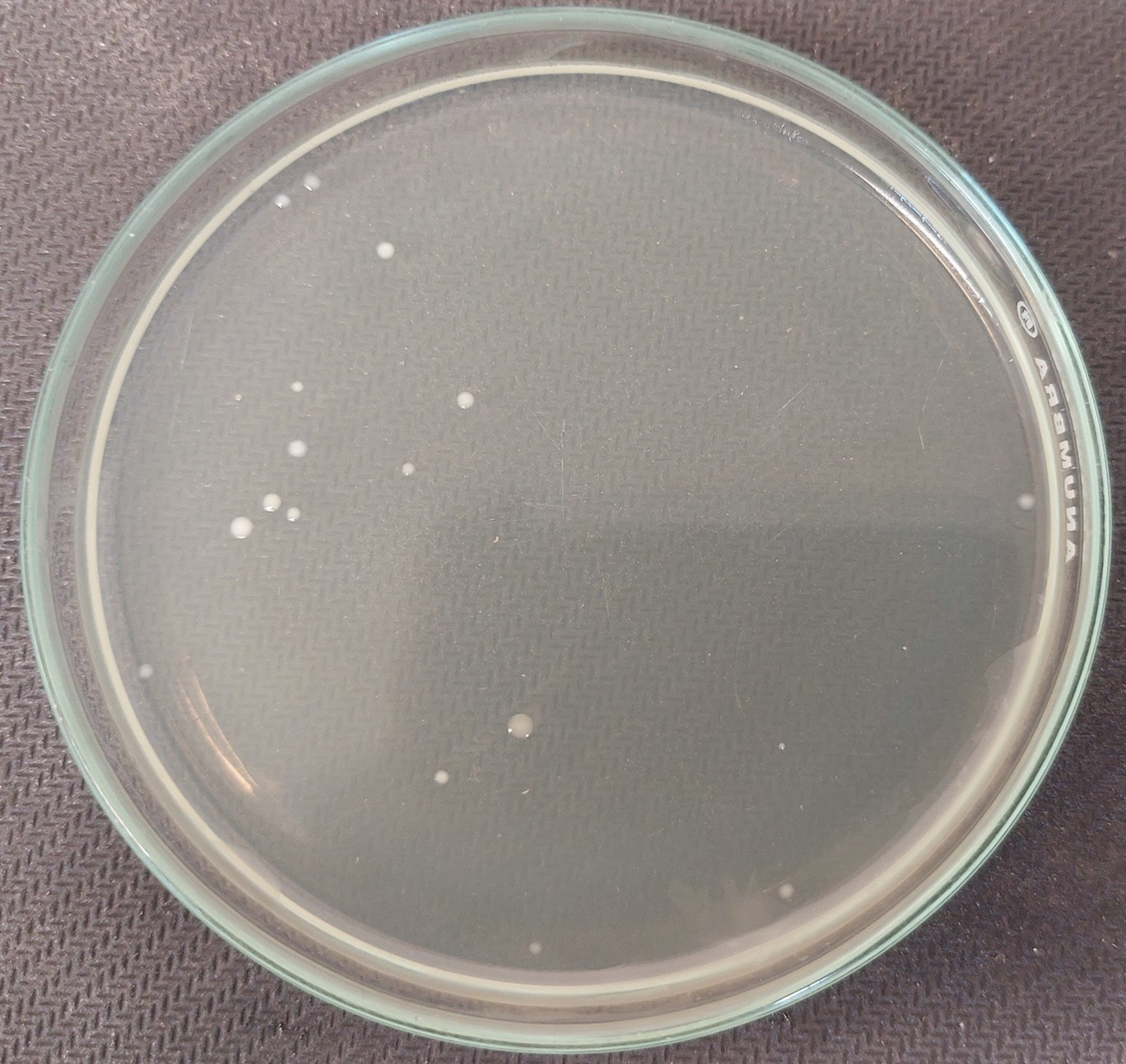

Supplement: S3 Fig — (TIFF) [file pone.0335979.s003.tiff]

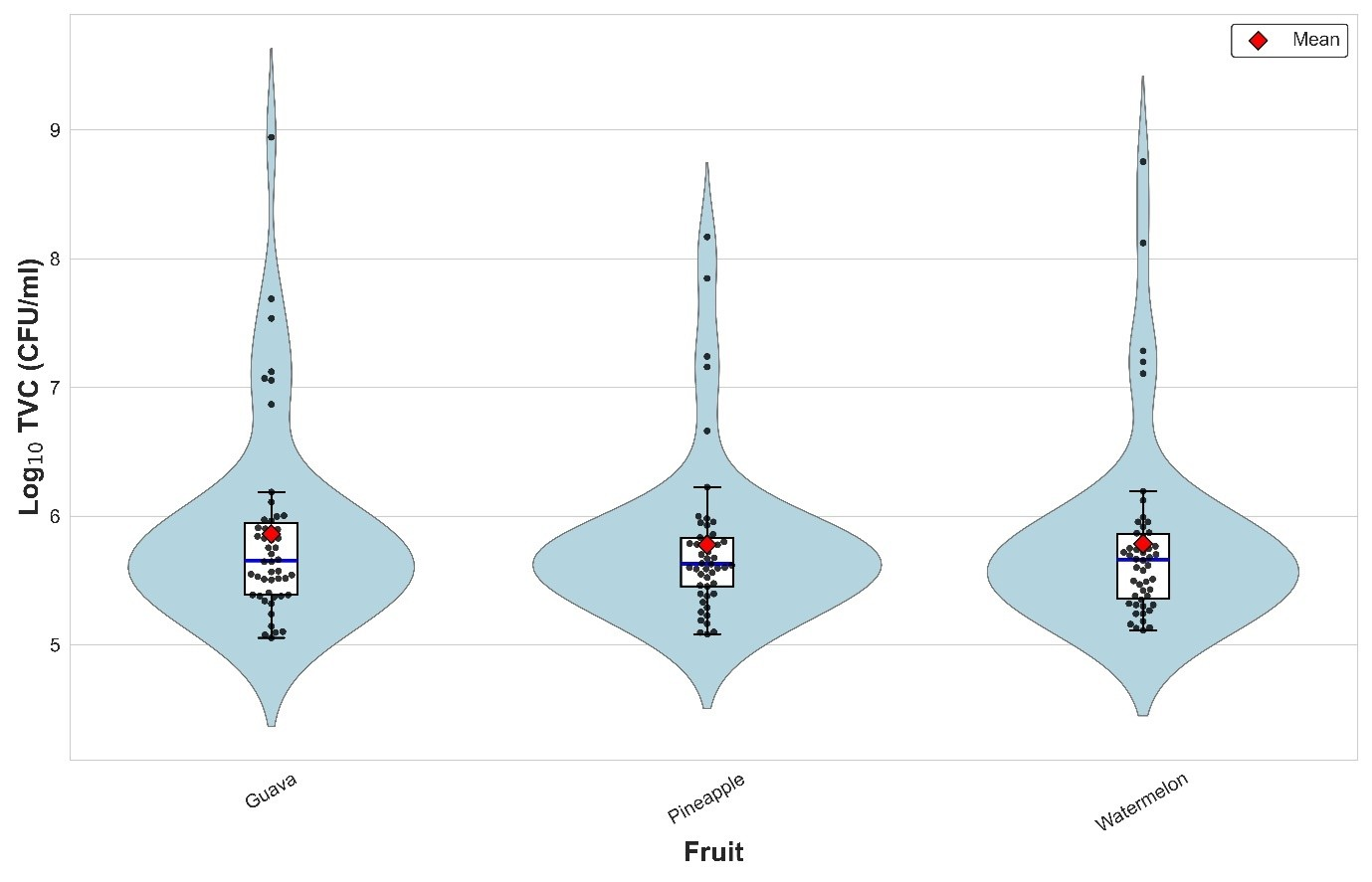

Supplement: S4 Fig — (TIFF) [file pone.0335979.s004.tiff]

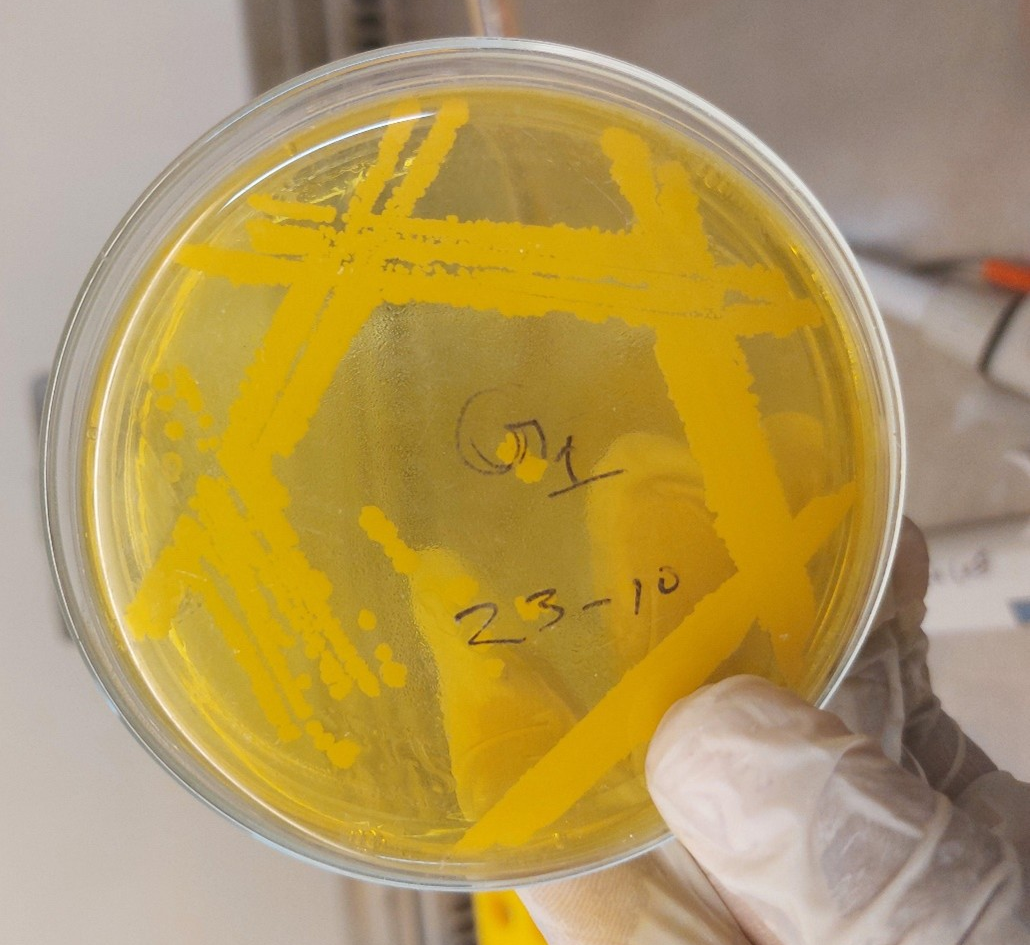

Supplement: S5 Fig — (TIFF) [file pone.0335979.s005.tiff]

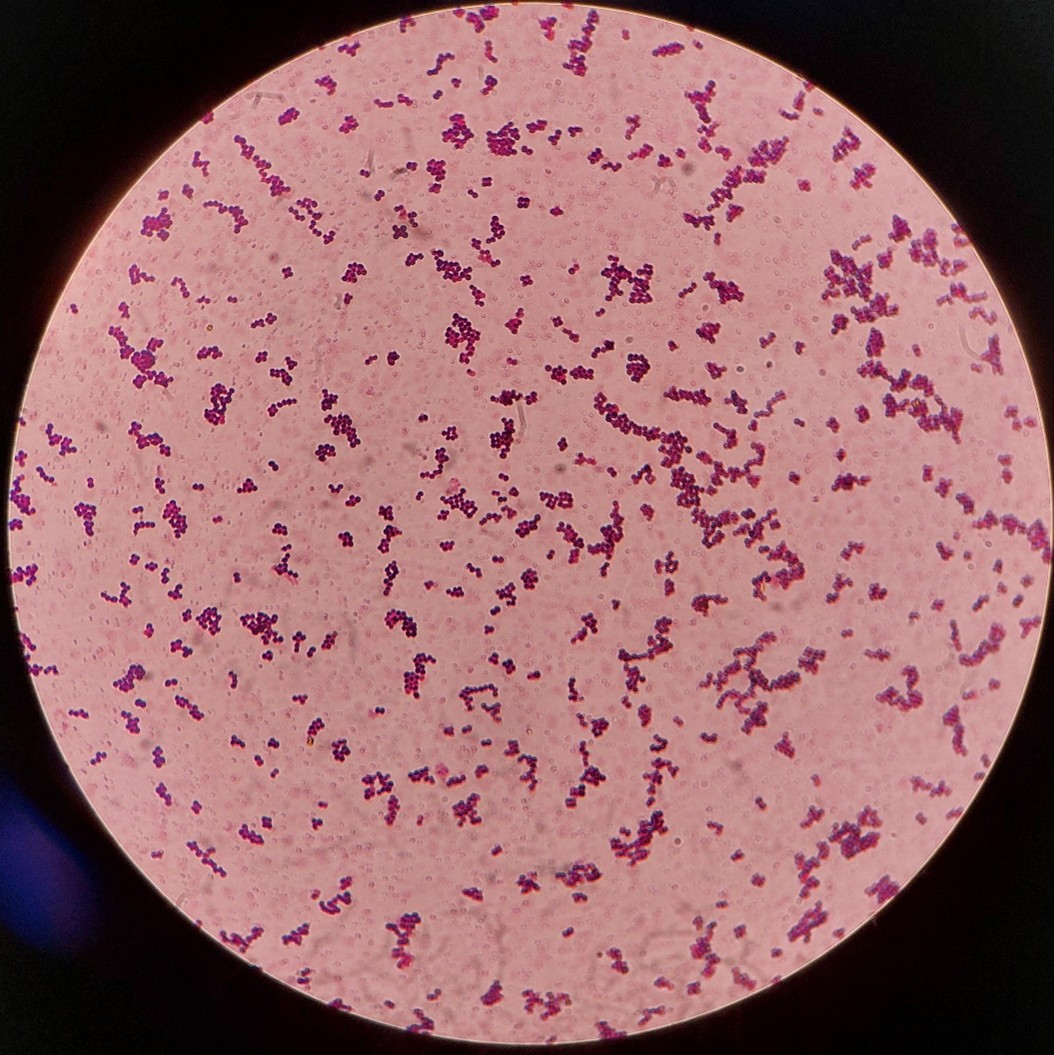

Supplement: S6 Fig — (TIFF) [file pone.0335979.s006.tiff]

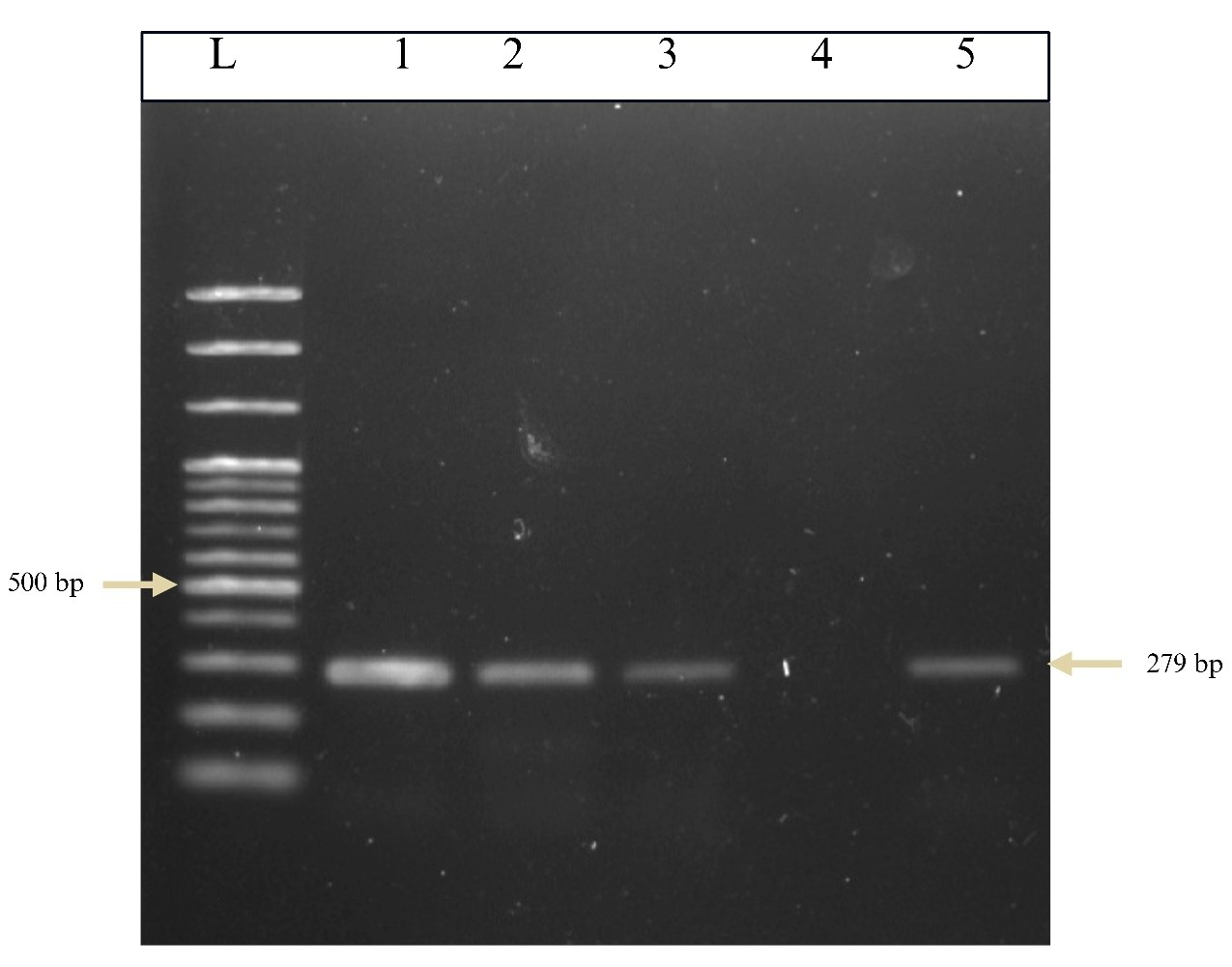

Supplement: S7 Fig — Lane L: 100 bp size DNA marker; Lane 1–3: DNA samples of S. aureus extracted from guava, pineapple, and watermelon; Lane 4: Negative control; Lane 5: Positive control. (TIFF) [file pone.0335979.s007.tiff]

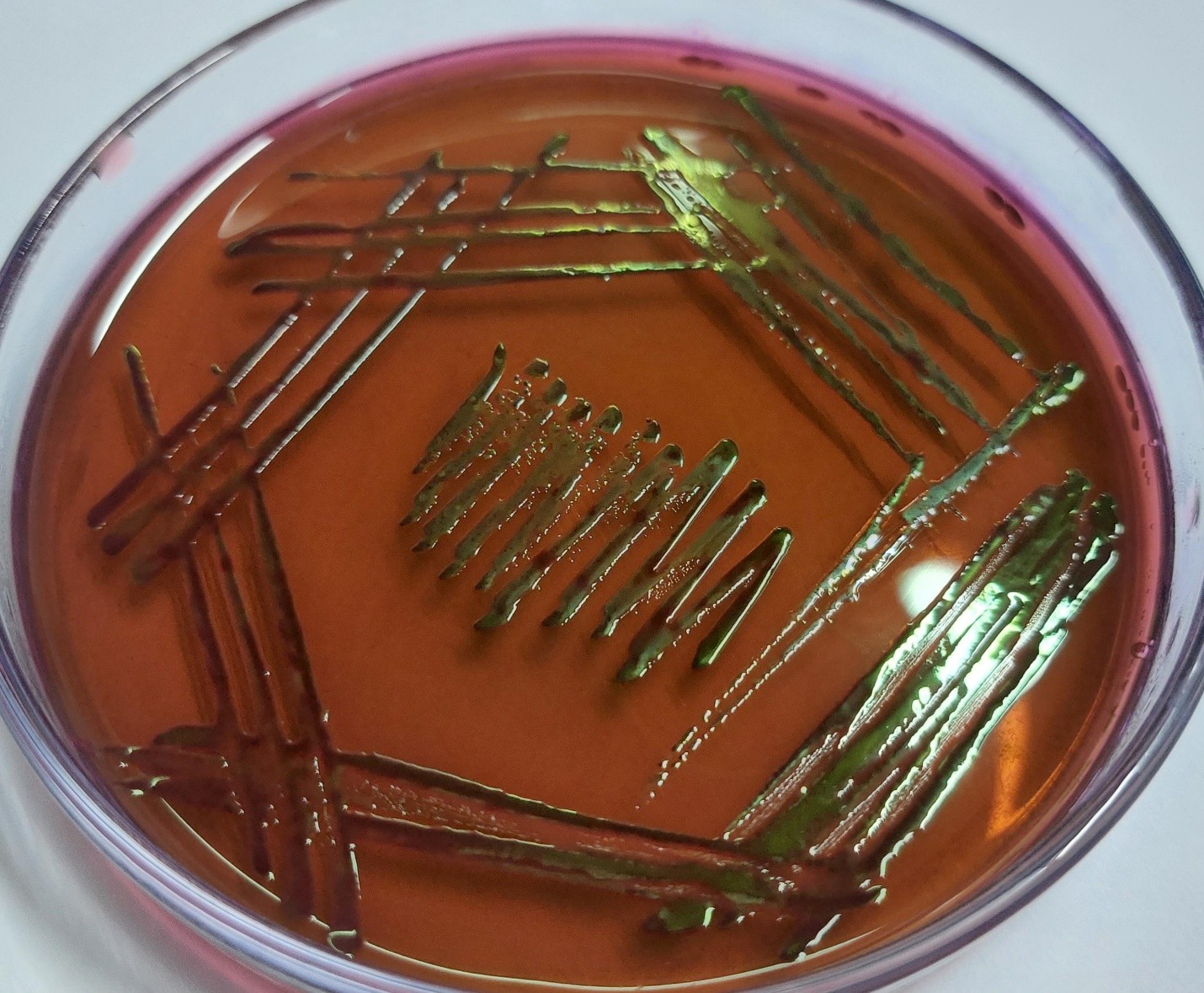

Supplement: S8 Fig — (TIFF) [file pone.0335979.s008.tiff]

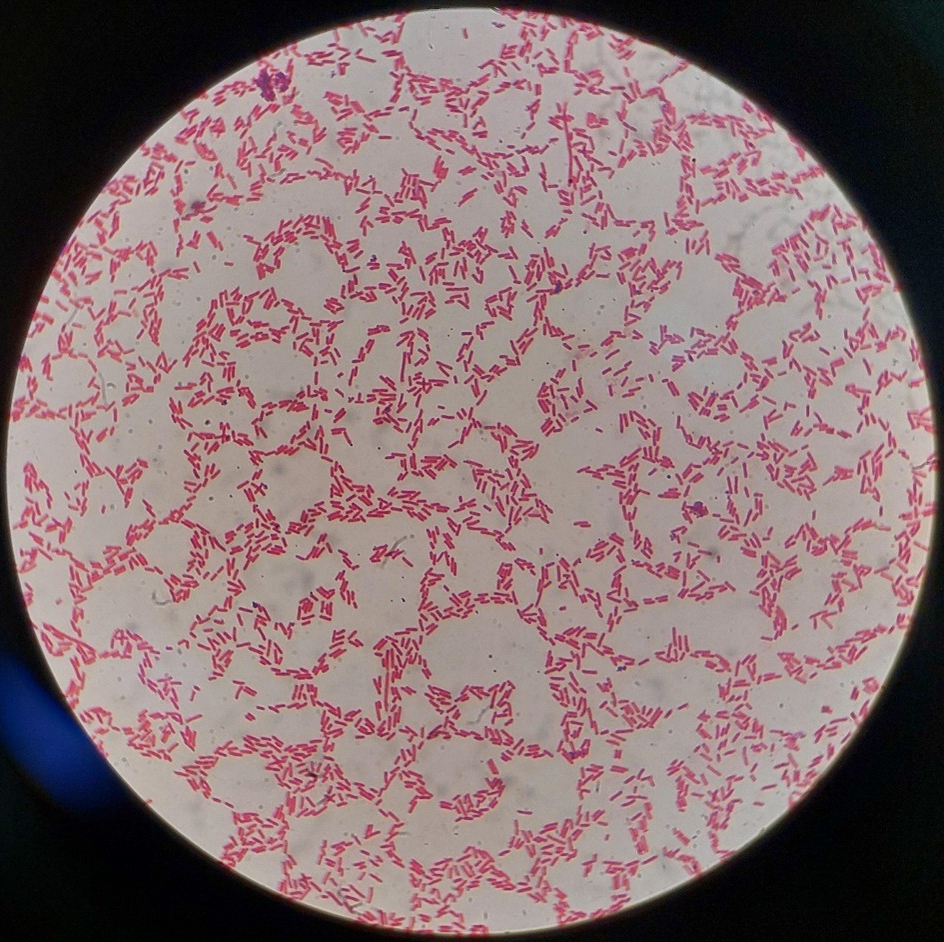

Supplement: S9 Fig — (TIFF) [file pone.0335979.s009.tiff]

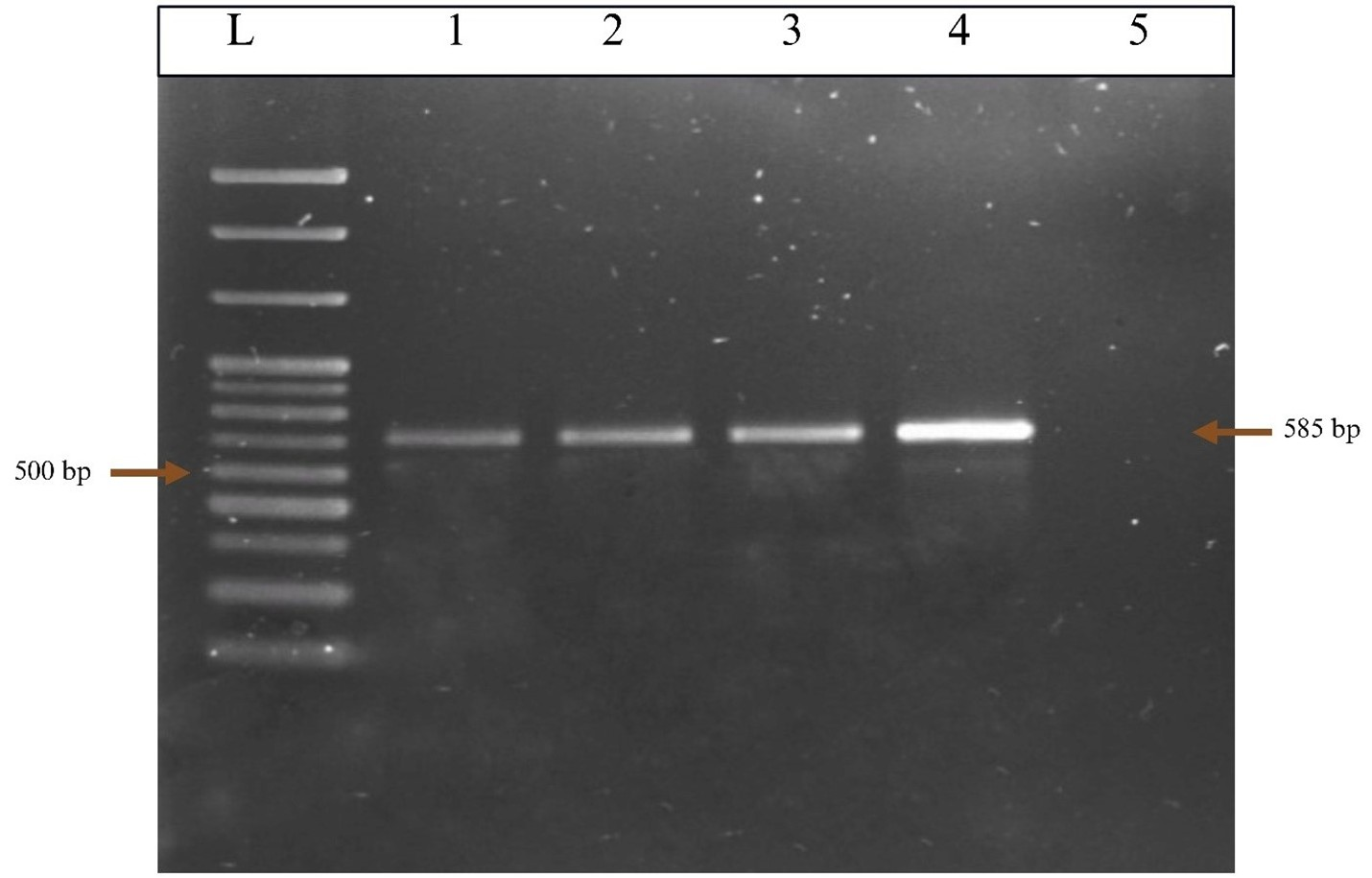

Supplement: S10 Fig — Lane L: 100 bp size DNA marker; Lane 1–3: DNA samples of E. coli extracted from guava, pineapple, and watermelon; Lane 3: Positive control. Lane 5: negative control. (TIFF) [file pone.0335979.s010.tiff]
