# Supplementary material for: Health effects of street vended fresh cut fruits: A randomized controlled trial in Bangladesh
Source: PLoS One. 2025 Oct 31;20(10):e0335979. doi: 10.1371/journal.pone.0335979 (PMC12578160; doi:10.1371/journal.pone.0335979)
Supplement: S1 Table — (DOCX) [file pone.0335979.s011.docx]

**Table S1.** Baseline demographics and hygienic scores among the participants.

| **Stratification** | **Subgroup** | **Count** | **Age Mean** | **Age STD** | **Age Minimum** | **AgeMaximum** | **HPS Mean** | **HPS STD** | **Amount of Fruit Mean** | **Amount of Fruit STD** |
| --- | --- | --- | --- | --- | --- | --- | --- | --- | --- | --- |
| Overall | All Participants | 300 | 28.61667 | 8.091227 | 18 | 57 | 39.21333333 | 3.039509436 |  |  |
| Group | Treatment | 150 | 28.56 | 8.278281 | 18 | 57 | 39.69333333 | 1.662643915 |  |  |
| Group + Sex | Treatment - Male | 75 | 28.86667 | 8.767775 | 19 | 57 | 39.70666667 | 1.674719285 |  |  |
| Group + Sex | Treatment - Female | 75 | 28.25333 | 7.805219 | 18 | 49 | 39.68 | 1.661650108 |  |  |
| Group | Control | 150 | 28.67333 | 7.927098 | 18 | 57 | 38.73333333 | 3.912922433 |  |  |
| Group + Sex | Control - Male | 81 | 28.80247 | 8.518245 | 18 | 57 | 38.91358025 | 3.362281706 |  |  |
| Group + Sex | Control - Female | 69 | 28.52174 | 7.230671 | 19 | 47 | 38.52173913 | 4.490541352 |  |  |
| Treatment Detailed | HAP - Guava - Male | 17 | 25.11765 | 6.469953 | 19 | 45 | 39.82352941 | 0.52859414 | 75.57470588 | 15.43513651 |
| Treatment Detailed | HAP - Guava - Female | 8 | 30.125 | 6.556077 | 21 | 39 | 39.875 | 0.353553391 | 83.40125 | 13.43961143 |
| Treatment Detailed | HAP - Pineapple - Female | 8 | 25.375 | 3.997767 | 21 | 34 | 38.375 | 4.596194078 | 71.39875 | 11.00262883 |
| Treatment Detailed | HAP - Pineapple - Male | 17 | 30.52941 | 10.93799 | 19 | 57 | 39.88235294 | 0.48507125 | 76.76117647 | 15.48710427 |
| Treatment Detailed | HAP - Watermelon - Male | 21 | 29.42857 | 7.527094 | 19 | 43 | 40 | 0 | 75.82714286 | 16.30919224 |
| Treatment Detailed | HAP - Watermelon - Female | 4 | 39.5 | 12.36932 | 21 | 47 | 40 | 0 | 72.5 | 16.01041328 |
| Treatment Detailed | ANAP - Guava - Female | 24 | 24.625 | 4.401704 | 18 | 37 | 39.75 | 1.032093693 | 73.92458333 | 14.52748878 |
| Treatment Detailed | ANAP - Guava - Male | 1 | 31 |  | 31 | 31 | 40 |  | 71 |  |
| Treatment Detailed | ANAP - Pineapple - Female | 16 | 29.75 | 8.940544 | 21 | 49 | 39.75 | 1 | 72.945 | 15.97557511 |
| Treatment Detailed | ANAP - Pineapple - Male | 9 | 30 | 11.05667 | 21 | 57 | 38.44444444 | 4.666666667 | 67.29 | 9.423620589 |
| Treatment Detailed | ANAP - Watermelon - Male | 10 | 30 | 8.640988 | 20 | 44 | 39.7 | 0.948683298 | 73.958 | 16.13408662 |
| Treatment Detailed | ANAP - Watermelon - Female | 15 | 30 | 8.42615 | 18 | 46 | 40 | 0 | 74.51866667 | 14.08089479 |

HPS: Hygienic Practice Score, STD: Standard Deviation.
