# Supplementary material for: Health effects of street vended fresh cut fruits: A randomized controlled trial in Bangladesh
Source: PLoS One. 2025 Oct 31;20(10):e0335979. doi: 10.1371/journal.pone.0335979 (PMC12578160; doi:10.1371/journal.pone.0335979)
Supplement: S2 Table — (DOCX) [file pone.0335979.s012.docx]

**Table S2.** Frequency of Symptoms among the participants.

| Group Type | Group Name | NSA (%) | VMN (%) | ACP (%) | WKS (%) | FVR | DRA (%) | BDRA | CSCF (%) | HBN (%) |
| --- | --- | --- | --- | --- | --- | --- | --- | --- | --- | --- |
| Intervention | Treatment | 13.33 | 6.67 | 8.67 | 10.67 | 6.00 | 4.67 | 1.33 | 8.00 | 8.67 |
| Intervention | Control | 1.33 | 1.33 | 1.33 | 3.33 | 1.33 | 0.00 | 0.00 | 0.67 | 4.67 |
| Fruit | Guava | 10.00 | 4.00 | 8.00 | 16.00 | 10.00 | 2.00 | 0.00 | 8.00 | 16.00 |
| Fruit | Pineapple | 18.00 | 10.00 | 6.00 | 6.00 | 6.00 | 4.00 | 4.00 | 6.00 | 4.00 |
| Fruit | Watermelon | 12.00 | 6.00 | 12.00 | 10.00 | 2.00 | 8.00 | 0.00 | 10.00 | 6.00 |
| Acidity | HAP | 14.67 | 5.33 | 9.33 | 13.33 | 9.33 | 5.33 | 1.33 | 10.67 | 14.67 |
| Acidity | ANAP | 12.00 | 8.00 | 8.00 | 8.00 | 2.67 | 4.00 | 1.33 | 5.33 | 2.67 |
| Age | 20-29 | 5.26 | 3.51 | 5.85 | 7.02 | 5.26 | 2.34 | 0.58 | 4.68 | 9.36 |
| Age | 30-39 | 10.71 | 3.57 | 2.38 | 4.76 | 1.19 | 2.38 | 1.19 | 3.57 | 4.76 |
| Age | <20 | 16.67 | 8.33 | 8.33 | 8.33 | 8.33 | 0.00 | 0.00 | 0.00 | 0.00 |
| Age | 40-49 | 3.57 | 3.57 | 7.14 | 10.71 | 0.00 | 3.57 | 0.00 | 3.57 | 0.00 |
| Age | 50+ | 20.00 | 20.00 | 0.00 | 20.00 | 0.00 | 0.00 | 0.00 | 20.00 | 0.00 |
| Sex | Male | 8.33 | 4.49 | 5.77 | 7.69 | 4.49 | 2.56 | 1.28 | 5.77 | 8.97 |
| Sex | Female | 6.25 | 3.47 | 4.17 | 6.25 | 2.78 | 2.08 | 0.00 | 2.78 | 4.17 |
