# Supplementary material for: Health effects of street vended fresh cut fruits: A randomized controlled trial in Bangladesh
Source: PLoS One. 2025 Oct 31;20(10):e0335979. doi: 10.1371/journal.pone.0335979 (PMC12578160; doi:10.1371/journal.pone.0335979)
Supplement: S3 Table — (DOCX) [file pone.0335979.s013.docx]

**Table S3.** Association between bacterial load (log_TVC) in the fruit samples and the Gastrointestinal Symptoms.

| **Symptom** | **Point-Biserial r (*p*-value)** | **Logistic Regression OR (95% CI, *p*)** | **Mann-Whitney U (*p*-value)** | **Adjusted *p*-value** |
| --- | --- | --- | --- | --- |
| NSA | 0.26 (0.001) | 2.16 (1.29–3.65, p=0.004) | U=2057 (p=0.027) | 0.035 |
| VMN | 0.35 (<0.001) | 3.35 (1.68–6.69, p<0.001) | U=939 (p=0.002) | 0.003 |
| ACP | 0.56 (<0.001) | 6.16 (2.87–13.23, p<0.001) | U=1588 (p<0.001) | <0.001 |
| WKS | 0.66 (<0.001) | 11.28 (4.48–28.43, p<0.001) | U=1860 (p<0.001) | <0.001 |
| FVR | 0.53 (<0.001) | 6.19 (2.64–14.54, p<0.001) | U=967 (p<0.001) | 0.003 |
| DRA | 0.51 (<0.001) | 6.73 (2.56–17.70, p<0.001) | U=715 (p=0.007) | 0.010 |
| BDRA | 0.06 (0.490) | 1.62 (0.40–6.61, p=0.500) | U=144 (p=0.954) | 0.954 |
| CSCF | 0.49 (<0.001) | 4.95 (2.40–10.21, p<0.001) | U=1215 (p=0.001) | 0.003 |
| HBN | 0.19 (0.020) | 1.94 (1.06–3.54, p=0.031) | U=1185 (p=0.050) | 0.056 |

NSA, nausea; VMN, vomiting; ACP, abdominal cramps and pain; WKS, weakness; FVR, fever; DRA, diarrhea; BDRA, bloody diarrhea; CSCF, change in stool consistency and frequency; HBN, heartburn.
