# Supplementary material for: Health effects of street vended fresh cut fruits: A randomized controlled trial in Bangladesh
Source: PLoS One. 2025 Oct 31;20(10):e0335979. doi: 10.1371/journal.pone.0335979 (PMC12578160; doi:10.1371/journal.pone.0335979)
Supplement: S4 Table — (DOCX) [file pone.0335979.s014.docx]

**Table S4.** Spearman and Pearson correlation between *S. aureus* and *E. coli* and GI symptoms after consumption of fruits.

| Symptom | Microbe | Spearman Rho | Spearman *p* value | Pearson r | Pearson *p* value | Spearman FDR *p* | *Pearson FDR p* |
| --- | --- | --- | --- | --- | --- | --- | --- |
| NSA | *S. aureus* | 0.451301 | 6.79E-09 | 0.451301 | 6.79E-09 | 1.53E-08 | 1.53E-08 |
| NSA | *E. coli* | 0.254478 | 0.001675 | 0.254478 | 0.001675 | 0.00201 | 0.00201 |
| VMN | *S. aureus* | 0.332792 | 3.17E-05 | 0.332792 | 3.17E-05 | 4.39E-05 | 4.39E-05 |
| VMN | *E. coli* | 0.476824 | 6.90E-10 | 0.476824 | 6.90E-10 | 2.07E-09 | 2.07E-09 |
| ACP | *S. aureus* | 0.465664 | 1.92E-09 | 0.465664 | 1.92E-09 | 4.93E-09 | 4.93E-09 |
| ACP | *E. coli* | 0.782595 | 2.86E-32 | 0.782595 | 2.86E-32 | 5.15E-31 | 5.15E-31 |
| WKS | *S. aureus* | 0.435249 | 2.60E-08 | 0.435249 | 2.60E-08 | 5.21E-08 | 5.21E-08 |
| WKS | *E. coli* | 0.694169 | 6.92E-23 | 0.694169 | 6.92E-23 | 6.12E-22 | 6.12E-22 |
| FVR | *S. aureus* | 0.332792 | 3.17E-05 | 0.332792 | 3.17E-05 | 4.39E-05 | 4.39E-05 |
| FVR | *E. coli* | 0.476824 | 6.90E-10 | 0.476824 | 6.90E-10 | 2.07E-09 | 2.07E-09 |
| DRA | *S. aureus* | 0.266808 | 0.000965 | 0.266808 | 0.000965 | 0.001241 | 0.001241 |
| DRA | *E. coli* | 0.692219 | 1.02E-22 | 0.692219 | 1.02E-22 | 6.12E-22 | 6.12E-22 |
| BDRA | *S. aureus* | 0.063605 | 0.439363 | 0.063605 | 0.439363 | 0.439363 | 0.439363 |
| BDRA | *E. coli* | 0.394215 | 5.99E-07 | 0.394215 | 5.99E-07 | 1.08E-06 | 1.08E-06 |
| CSCF | *S. aureus* | 0.357983 | 6.87E-06 | 0.357983 | 6.87E-06 | 1.12E-05 | 1.12E-05 |
| CSCF | *E. coli* | 0.671177 | 5.55E-21 | 0.671177 | 5.55E-21 | 2.50E-20 | 2.50E-20 |
| HBN | *S. aureus* | 0.249579 | 0.00207 | 0.249579 | 0.00207 | 0.002329 | 0.002329 |
| HBN | *E. coli* | 0.171193 | 0.036205 | 0.171193 | 0.036205 | 0.038334 | 0.038334 |
